# Construction of an immune-related ceRNA network in cervical cancer based on HPV E6 splicing

**DOI:** 10.3389/fonc.2022.979884

**Published:** 2022-12-14

**Authors:** Senwei Jiang, Yun Zhou, Minjuan Ye, Xiaomao Li, Lan Zhang, Yuebo Yang

**Affiliations:** ^1^ Department of Gynecology, The Third Affiliated Hospital of Sun Yat-sen University, Guangzhou, China; ^2^ Department of Gynecology, State Key Laboratory of Oncology in South China, Collaborative Innovation Center for Cancer Medicine, Sun Yat-Sen University Cancer Center, Guangzhou, China; ^3^ Department of Radiation Oncology, The Third Affiliated Hospital of Kunming Medical University (Yunnan Cancer Hospital, Yunnan Cancer Center), Kunming, China

**Keywords:** cervical cancer, HPV, E6, alternative splice, immunotherapy, ceRNA Network, prognosis, immune infiltration

## Abstract

**Background:**

Cervical cancer is one of the leading causes of cancer-related deaths worldwide. The unspliced human papillomavirus (HPV) E6 plays an important role in tumor progression and immune regulation. Improved immunotherapy implementation might benefit from a better knowledge of HPV E6 splicing-related immune gene expressions and immunocyte infiltration in cervical cancer. This study aimed to identify the potential therapeutic and prognostic roles of unspliced/spliced E6 ratio (E6 ratio) in cervical cancer.

**Methods:**

Data from the TCGA were used to analyze the E6 condition and clinical information. Nomogram and K-M analysis were used to analyze assess the prognostic significance, IOBR was used to investigate immunological infiltrates. Functions and pathway enrichment analysis of DEGs were investigated through GO analysis and KEGG pathway analysis, respectively. A core module was taken from the competitive endogenous RNA (ceRNA) network and used to build a lncRNA-miRNA-mRNA network. QT-qPCR was used to detect the expression of genes. CCK-8, colony formation, wound healing and migration assays were used to detect cell functions.

**Results:**

Our study found that HPV E6 ratio had significantly correlation with overall survival. In cervical cancer, a high E6 ratio was adversely linked with infiltrating levels of aDC, M1 macrophages, monocytes, NKT, and Tgd. High E6 ratio phenotypes were shown to be implicated in immune response regulation, cell adhesion, and Wnt signaling pathways, according to functional enrichment analysis. Subsequently, we constructed an immune-related ceRNA network based on E6 splicing in cervical cancer, including three lncRNA (*LINC00943, LIFR-AS1, DANT2*, and *RASSF8-AS1*), four miRNA (miR-205-5p, miR-181d-5p, miR-222-3p, and miR-221-3p), and seven mRNA (*FGFR1*, *PRLR*, *CXCL2*, *ISG20*, *ISG15*, *SDC1*, and *NR2F2*). Among them, *CXCL2*, *SDC1*, and miR-221-3p were associated with survival and immune cell infiltration.

**Conclusions:**

These data imply that a high E6 ratio in cervical cancer contributes to the immune-related ceRNA network, resulting in a low amount of infiltrating effector immune cells and tumor growth. As a result, the E6 ratio might be employed as a biomarker in cervical cancer to determine prognosis and treatment success.

## Introduction

Cervical cancer is the most deadly gynecologic malignancy worldwide, with an estimated 604,000 new cases and 342,000 deaths globally in 2020 (1). Although the incidence and mortality of cervical cancer in developed countries have gradually declined with human papillomavirus (HPV) vaccination, the rates have elevated rapidly in developing countries such as Eastern Africa, Southern Africa, and Middle Africa (2). Around 90% of cervical cancer fatalities occurred in low- and middle-income countries, where mortality is 18 times that of developed ones (3). Deaths from cervical cancer can be prevented through HPV vaccination, regular screening, and early treatment (4). In 2020, the World Health Organization approved a 90-70-90 strategy aimed at eliminating cervical cancer worldwide, with 90% of women diagnosed with cervical disease receiving treatment (5).

Treatment options and results for cervical cancer patients are greatly dependent on the disease stage at diagnosis, with 5-year survival rates ranging from over 90% in early, localized stages to fewer than 20% in distant or metastatic stages (6). Radical hysterectomy with pelvic lymphadenectomy, chemotherapy, and radiation therapy are all alternatives for early-stage cervical cancer treatment (7). For advanced cervical cancer, first-line cisplatin-based chemotherapy remains the best choice but is rarely curative (8). Immunotherapies, particularly checkpoint inhibitor therapy, have gained popularity as a treatment option for advanced malignancies. The use of immunotherapy to treat cervical cancer is currently being researched; however, the US Food and Drug Administration (FDA) has only licensed one immune checkpoint inhibitor (pembrolizumab) for use against cervical cancer (6).

Persistent infections with high-risk human papillomavirus (HR-HPV) lead to almost all cervical cancers (5). HPV16, 18, 31, 33, 35, 39, 45, 51, 52, 56, 58, 59, 68, 73, and 82 are the 15 HPV types most typically discovered in cancer biopsies and are thus categorized as HR-HPV (9). Over 85% of cervical cancer cases are caused by high-risk kinds, with HPV16 and HPV18 being the most frequent high-risk forms (10). The HPV genome encodes E1, E2, E4, E5, E6, and E7, oncoproteins that inhibit tumor suppressors p53 and pRb. E6 and E7 are particularly essential since they are oncoproteins that repress tumor suppressors p53 and pRb (6). The carcinogenic E6 and E7 genes are frequently integrated and expressed when HPV integrates into the host genome during advancement, whereas the other HPV genes are deleted or not expressed (11). Alternative splicing is a vital post-transcriptional regulatory mechanism in the development of malignancies, as well as one of the most important post-transcriptional regulatory processes in the development of cancers (12). HR-HPV has a common trait of alternative splicing within E6-E7 open reading frames (ORF), and full-length E6 from HR-HPV types is produced from mRNA with no splicing within E6 ORF (13). Spliced-E6 (E6*) is found in larger levels in premalignant and malignant cervical and oropharyngeal lesions positive for HPV16 genomes than unspliced-E6 (E6) (9). The roles of E6 spliced isoforms are mostly unknown at this time. E6* is speculated to be involved in tumor growth, apoptosis regulation, angiogenesis, and cell polarity and adhesion (14). Although HPV promotes tumor progression by evading the immune system, the immunomodulatory function of E6 has not yet been thoroughly studied.

Because HPV infection is responsible for nearly all cases of cervical cancer, a better understanding of molecular biology and tumor-host immune system interactions has led to the development of promising new targeted therapeutic modalities that can be added to the currently limited arsenal of effective cervical cancer treatments (15). Therefore, to improve the effectiveness of immunotherapy for patients with cervical cancer, we aimed to identify key genes and pathways that may be related to the phenotypic diversity of patients with E6 and E6*. Here, we explored the immune-related competing endogenous RNA (ceRNA) network on E6 splicing in cervical cancer. Our results provide a new understanding of the mechanisms and potential therapeutic targets for cervical cancer.

## Materials and methods

### Data source

The lncRNA/microRNA/mRNA expression data and clinical information of cervical cancer patients were downloaded from The Cancer Genome Atlas (TCGA) (https://cancergenome.nih.gov). The HPV infection staus, HPV variants and E6 unspliced/spliced transcripts were downloaded form TCGA (16). The following samples were excluded: (1) repeated sequencing results; (2) insufficient survival information; (3) insufficient HPV E6 splicing data. A total of 126 cervical cancer patients with complete clinical information (ie, age, sex, primary tumor site, metastatic state at diagnosis, survival time, and survival state) and HPV data (ie, HPV type, E6 unspliced normalized counts [E6], E6 spliced normalized counts [E6spl], E6 unspliced/spliced ratio [E6ratio], primary HPV integration variable and secondary HPV integration variable) were included in our analysis. Follow-up and expression profiles were processed as we previously described (17).

### Nomogram analysis

Regression Modeling Strategies (rms) is a collection of functions that assist with and streamline modeling (18). In this study, we used the R package rms, integrating data on survival time, survival status and 11 characteristics, and built a nomogram using the cox method to assess the prognostic significance of these characteristics.

### Immune cells infiltration abundance analysis

IOBR (Immuno-Oncology Biological Research) is a computational tool for immuno-tumor biology research (19). Here, based on our expression profiles, we use IOBR package in R to analysis the immune cells infiltration abundance of 126 cervical cancer patients. xCell method was select to calculate infiltration abundance of 64 kinds of immune cells, stem cells and stromal cells in each sample (20). The DEGs of ceRNA Network were divided to the high expression and low expression groups by median. The relationship between DEG expression and the fractions of immune cells was investigated by Wilcoxon test.

### Identification of DEGs

The Linear Models for Microarray Analysis (Limma) package in R software was applied to identify differentially expressed genes (DEGs) in the high E6ratio cervical cancer compared with the E6ratio cervical cancer, based on Student’s t-test (21). Adjusted p-values were calculated using the Benjamini-Hochberg method. The significant DEGs were selected with a threshold of p-value <0.05 and fold change>1.5. We got volcano plot utilizing the pheatmap package in R (22).

### Functional enrichment analysis

In order to explore potential functions and pathways that may be altered by the DEGs, we applied the clusterProfiler package in R to perform functional and pathway enrichment analyses of the identified DEGs (23). The Gene Ontology (GO; http://www.geneontology.org/) database was used to determine the biological processes (BPs) that the DEGs may be involved. In addition, according to the modified Fisher’s exact test, the Kyoto Encyclopedia of Genes and Genomes (KEGG; http://www.genome.jp/kegg/pathway.html) database was used for pathway enrichment analysis of the identified DEG. The selection criteria for significant GO terms and pathways were p <0.05, and the number of enriched genes (also called count)> 2.

### Gene set enrichment analysis

GSEA software (version 3.0) and c2.all.v7.4.symbols.gmt sub-collection were obtained from the GSEA website (http://software.broadinstitute.org/gsea/index.jsp) (24). We divided the samples into two groups by E6ratio. The minimum gene set was 5 and the maximum gene set was 5000, with 1000 resampling, p-value < 0.05 (as needed) or FDR < 0.25 (as needed) were considered statistically significant.

### Construction of ceRNA network

Immune genes list was obtained from the Immunology Database and Analysis Portal (IMMPORT) database (http://www.immport.org/) (25). The Venny online tool was used to analyze overlapping genes between immune genes and DE-mRNAs (http://jvenn.toulouse.inra.fr/) (26). The miRNet database (https://www.mirnet.ca/) is an integrated platform tool for miRNA-associated studies (27). “Organism-H.sapies”, “Tissue-Cervix” and “target type-lncRNAs, miRNAs, Genes” were set as selection criteria. Using the miRNet database, we confirmed the DElncRNAs-DEmiRNAs interactions and DEmiRNAs-DEmRNAs interactions., which is an linking miRNAs, targets, and functions, Finally, we constructed a ceRNA network using Cytoscape software (version 3.8.1). In addition, we extracted a sub-network by cytoHubber plug-in. Furthermore, with the pearson correlation of these genes in TCGA-CESC, we identified E6 splicing Immune-related 14 hub genes.

### Predicted PPI network analysis

A Protein-Protein Interaction (PPI) network composed of 50 ceRNA network co-expression proteins was constructed by GeneMANIA (http://genemania.org/) (28). These nodes represent genes that are closely related to the ceRNA network in terms of physical interactions, shared protein domains, predictions, co-localization, pathway, co-expression, and genetic interactions.

### Cell culture and cell transfection

The human cancer cell lines SiHa and Caski were obtained from the Cell Bank, Shanghai Institutes for Biological Sciences (Shanghai, China). Cells were grown in a DMEM medium (Gibco BRL, San Francisco, CA, USA), supplemented with a 10% fetal bovine serum (Gibco BRL, San Francisco, CA, USA).

The pCDNA3.1(+)-E6 and pCDNA3.1(+)-E6* plasmids, as well as their control plasmids were purchased from IGE BIO (Guangzhou, China); the HPV16-E6 and HPV16-E6* sequences from NCBI are summarized in **Supplementary Table S5**. For transfection, 2ug of plasmids were dissolved in 250ul of Opti-mem media (Life Technology). The Lipofectamine 3000 reagent (Life Technology) was used to achieve cell transfection in accordance with the manufacturer’s instructions.

### RNA extraction and quantitative real-time PCR

The total RNA from cultured cells was extracted using the Trizol reagent (Life Technology) in accordance with the manufacturer’s instructions. Reverse transcriptase reactions using MMLV reverse transcriptase reagents (ES Science, China) were performed following the manufacturer’s protocol. Gene expression levels were normalized to house-keeping genes GAPDH. Reactions were performed in triplicate with the Roche LightCycler 480 II PCR system (Roche Diagnostics, Rotkreuz, Switzerland). Primer sequences are listed in **Supplementary Table S5**.

### Cell counting kit-8 and colony-formation assays

To assess the vitality of the cells, we employed a commercial tool called CCK-8 (HUAYUN, China). 96-pore dishes were seeded with cells at a density of 5*10^3^ cells per well, and an additional 10 ul of CCK-8 solution was added for an additional two hours of growth. The absorbance of each well was found to be 450 nm.

For colony-formation assays, cells were resuspended in medium containing 10% FBS and seeded into 6-well plates (1000 cells/well). The plates were cultured for 7 days and colonies with >50 cells were counted. Cell colony were stained with 4% paraformaldehyde and crystal violet, air dried and photographed.

### Wound healing and migration assays

For wound healing assays, The celllines were plated to confluence in 6-well plates. Scratches were made in the monolayer with a pipette tip. The progression of cell migration was observed and photographed at 24h after wounding. The cell migration assays were performed using transwell chambers (BD, Durham, NC, USA). A medium containing 10% FBS was added to the lower chamber as a chemoattractant. After 24h incubation, migration cells located on the lower side of the chamber were stained with 4% paraformaldehyde and crystal violet, air dried and photographed.

### Statistical analysis and plots processing

Wilcoxon signed-rank test and logistic regression were performed to analyze the association between clinical features and E6ratio in cervical cancer. Kaplan-Meier analysis was performed to draw survival curves. Univariate Cox analysis to screen potential prognostic factors, and multivariate Cox analysis to verify the prognostic factors. All statistical analyses were performed using SPSS software (version 19.0) and R (version 3.6.4), a p-value less than 0.05 is considered as statistically significant. The plots were performed by R (version 3.6.4), Cytoscape (version 3.8.1). and SangerBox tools (version 3.0, http://www.sangerbox.com/tool).

## Results

### Correlation between E6 splicing and clinical features in cervical cancer

The RNA sequence data for a total of 126 patients with cervical cancer were acquired from the TCGA dataset. The detailed baseline clinical features are presented in **Table 1**. Among the 126 participants, 75 had a low unspliced/spliced E6 ratio (E6 ratio, E6/E6*) and 51 had a high E6 ratio. The E6 ratio (low vs. high) of patients with cervical cancer was significantly correlated with pathologic grade (*p* = 1.61E-02), overall survival (OS, 1315.00 ± 1308.12 days vs. 883.73 ± 985.42 days, *p* = 4.80E-02), progression-free survival (PFS, 1251.32 ± 1294.41 days vs. 761.98 ± 869.21 days, *p* = 2.70E-02), cancer status (*p* = 5.00E-02), HPV type (*p* = 7.80E-08), E6 unspliced normalized counts (E6, 25.75 ± 16.42 vs. 38.15 ± 16.18, *p* = 5.34E-05), E6 spliced normalized counts (E6*, 72.27 ± 58.73 vs. 41.92 ± 18.99, *p* = 5.24E-04), and E6 ratio (0.38 ± 0.13 vs. 1.01 ± 0.45, *p* = 3.30E-21). Other clinical features such as age, height, weight, body mass index (BMI), histological type, tumor-node-metastasis (TNM) stage, International Federation of Gynecology and Obstetrics (FIGO) stage and primary and secondary HPV integration variables were not significantly correlated with the E6 ratio.

**Table 1:** Correlations between E6ratio and clinicopathological features of patients with cervical cancer.

Characteristics	E6ratio_low (N=75)	E6ratio_high (N=51)	Total (N=126)	*p*-value
Age				9.57E-01
Mean±SD	45.61±11.94	45.49±13.13	45.56±12.38	
Height				9.62E-01
Mean±SD	162.83±6.71	162.76±7.56	162.81±6.99	
Weight				9.34E-01
Mean±SD	74.07±18.55	74.35±15.38	74.18±17.35	
BMI				7.92E-01
Mean±SD	28.20±6.56	27.87±5.35	28.08±6.14	
Histological_type				9.00E-02
ADC	11 (8.73%)	12 (9.52%)	23 (18.25%)	
ADSQCC	0 (0.0e+0%)	2 (1.59%)	2 (1.59%)	
SQCC	64 (50.79%)	37 (29.37%)	101 (80.16%)	
TNM_T				5.40E-01
T1	38 (37.25%)	30 (29.41%)	68 (66.67%)	
T2	15 (14.71%)	9 (8.82%)	24 (23.53%)	
T3	1 (0.98%)	0 (0.0e+0%)	1 (0.98%)	
T4	1 (0.98%)	0 (0.0e+0%)	1 (0.98%)	
TX	3 (2.94%)	5 (4.90%)	8 (7.84%)	
TNM_N				6.80E-01
N0	34 (33.33%)	22 (21.57%)	56 (54.90%)	
N1	18 (17.65%)	16 (15.69%)	34 (33.33%)	
NX	6 (5.88%)	6 (5.88%)	12 (11.76%)	
TNM_M				6.40E-01
M0	28 (28.00%)	20 (20.00%)	48 (48.00%)	
M1	1 (1.00%)	0 (0.0e+0%)	1 (1.00%)	
MX	28 (28.00%)	23 (23.00%)	51 (51.00%)	
FIGO_stage				7.00E-02
I	42 (33.87%)	40 (32.26%)	82 (66.13%)	
II	14 (11.29%)	5 (4.03%)	19 (15.32%)	
III	13 (10.48%)	6 (4.84%)	19 (15.32%)	
IV	4 (3.23%)	0 (0.0e+0%)	4 (3.23%)	
Pathologic_grade				**1.61E-02**
G1+G2	50 (39.69%)	23 (18.25%)	13 (57.94%)	
G3+GX	25 (19.84%)	28 (22.22%)	53 (42.07%)	
OS				**4.80E-02**
Mean±SD	1315.00±1308.12	883.73±985.42	1140.44±1202.75	
Status				2.00E-01
Alive	64 (50.79%)	38 (30.16%)	102 (80.95%)	
Dead	11 (8.73%)	13 (10.32%)	24 (19.05%)	
PFS				
Mean±SD	1251.32±1294.41	761.98±869.21	1066.28±1172.62	**2.70E-02**
Cancer_Status				**5.00E-02**
Not Available	7 (5.56%)	9 (7.14%)	16 (12.70%)	
Tumor free	58 (46.03%)	29 (23.02%)	87 (69.05%)	
With tumor	10 (7.94%)	13 (10.32%)	23 (18.25%)	
HPV type				**7.80E-08**
HPV16	72 (57.14%)	28 (22.22%)	100 (79.37%)	
HPV18	3 (2.38%)	23 (18.25%)	26 (20.63%)	
E6 unspliced normalized counts [E6]				**5.34E-05**
Mean±SD	25.75±16.42	38.15±16.18	30.77±17.37	
E6 spliced normalized counts [E6*]				**5.24E-04**
Mean±SD	72.27±58.73	41.92±18.99	59.98±49.09	
Primary HPV integration variable				9.20E-01
No	15 (11.90%)	9 (7.14%)	24 (19.05%)	
Yes	60 (47.62%)	42 (33.33%)	102 (80.95%)	
Secondary HPV integration variable				3.70E-01
No	26 (20.63%)	13 (10.32%)	39 (30.95%)	
Yes	49 (38.89%)	38 (30.16%)	87 (69.05%)	

Bold values indicate *P*<0.05.

**Figure 1:**
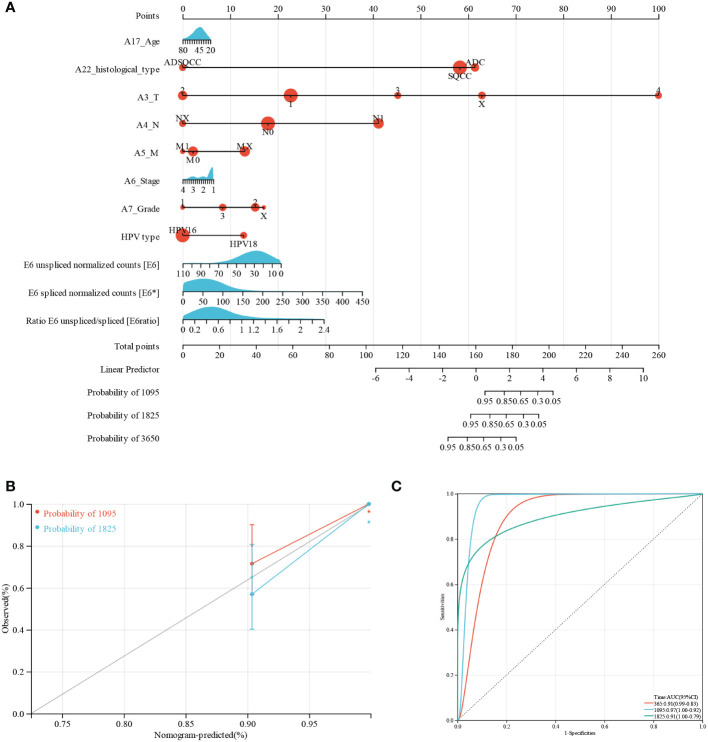
Predictive nomogram construction and validation. **(A)** A nomogram for predicting the 3-, 5-, and 10-year OS of patients with CESC. **(B)** Nomogram calibration curves for OS prediction at 3 and 5 years. **(C)** ROC analysis of nomogram.

Furthermore, we performed nomogram analysis to predict the possibility of 3- and 5-year OS. We integrated the prognostic characteristics of E6, E6*, E6 ratio, and other clinicopathological factors, including age, histological type, pathologic grade, FIGO stage, HPV type, and TNM stages. The score awarded to each component is proportionate to its risk contribution to survival, as illustrated in **Figure 1A**. The calibration curve’s indication corresponds nicely (**Figure 1B**). The areas under the receiver operating characteristic curve after one, three, and five years were 0.91, 0.97, and 0.91, respectively (**Figure 1C**).

### E6 splicing is associated with survival in cervical cancer

**Figure 2:**
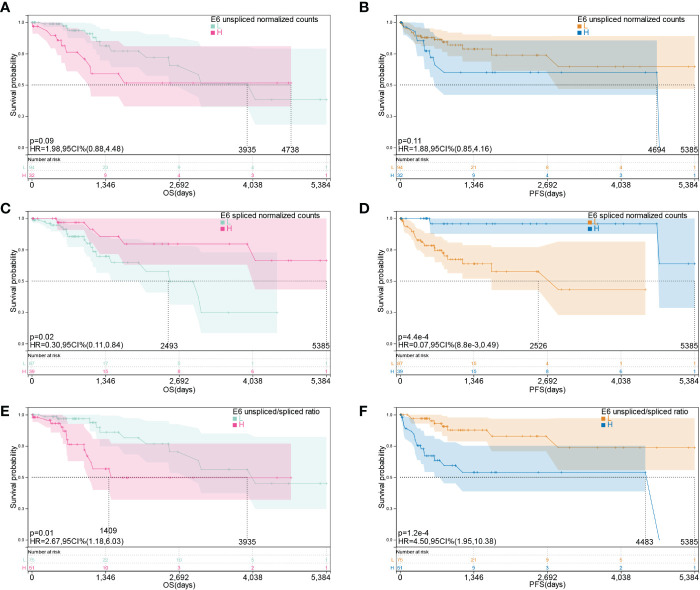
E6 splicing is associated with survival outcome in cervical cancer. **(A, B)** Overall survival and disease-free survival of E6 unspliced normalized counts (E6) in TCGA- CESC cohort. **(C, D)** Overall survival and disease-free survival of E6 spliced normalized counts (E6*) in TCGA-CESC cohort. **(E, F)** Overall survival and disease-free survival of unspliced/spliced E6 ratio (E6ratio) in TCGA-CESC cohort.

Moreover, we investigated the association between E6 splicing and clinical outcome features in TCGA-CESC. The connection between E6 splicing and the survival outcomes of TCGA-CESC populations was investigated using Kaplan–Meier survival analysis. The E6 unspliced normalized counts showed no significant correlation with OS (hazard ratio [HR] = 0.88–4.48, *p* = 0.09) and PFS (HR = 0.85–4.16, *p* = 0.11) (**Figures 2A, B**). The low E6* group had significantly shorter OS (HR = 0.11–0.84, *p* = 0.02) and PFS (HR = 0.009–0.49, *p* = 4.4E-4) than the high E6* group (**Figures 2C, D**). Moreover, E6 ratio showed a significant negative correlation with OS (HR = 1.18-6.03, *p* = 0.01) and PFS (HR = 1.95–10.38, *p* = 1.2E-4) (**Figures 2E, F**).

### E6 ratio is correlated with immune infiltration levels in cervical cancer

**Figure 3:**
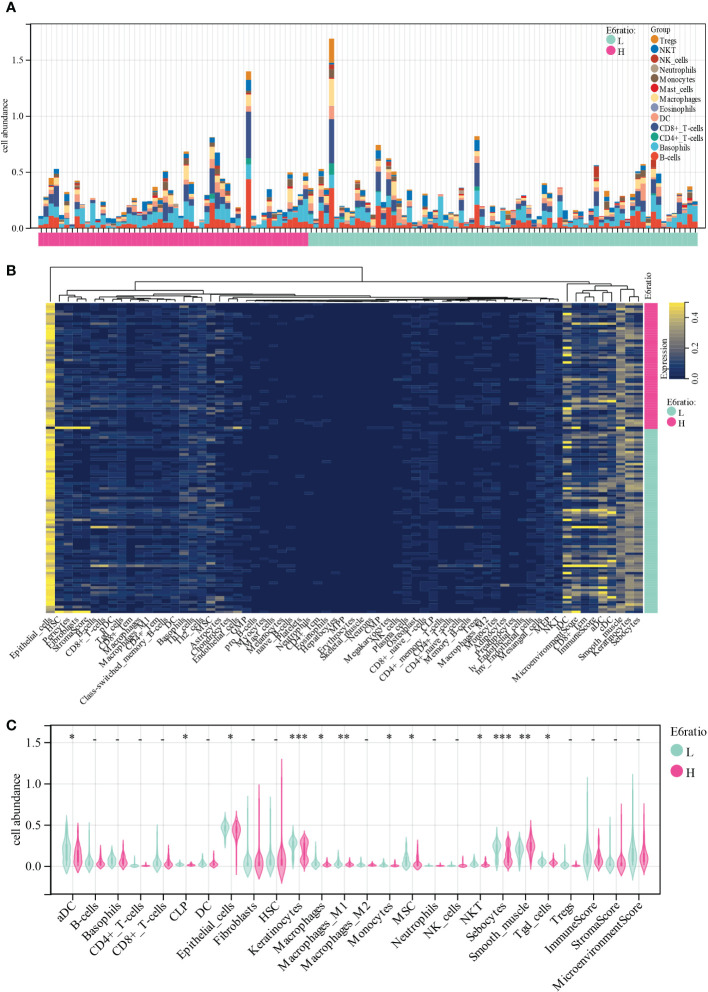
Correlations of E6ratio with immune infiltration level in CESC. **(A)** Distribution of immune cell infiltration in each sample. **(B)** Heatmap of immune cell types. **(C)** Violin plot of infiltrating immune cells in low and high E6ratio group. *, p < 0.05; **, p < 0.01; ***, p < 0.001.

To gain insights into the potential target immune cells for treating cervical cancer with immunotherapy, we estimated the composition of the microenvironment in patients in the TCGA-CESC cohort using the xCell algorithm. The composition of the microenvironment of cervical cancer was found to be complex (**Figure 3A**). The top five abundant cell types were epithelial cells, sebocytes, keratinocytes, smooth muscle, and immature dendritic cells (**Figures 3B**). Compared with patients with high E6 ratio, patients with low E6 ratio had a significant positive correlation with an abundance of activated dendritic cells (aDC, *p* = 0.01), common lymphoid progenitor (CLP, *p* = 0.02), epithelial cells (*p* = 0.03), keratinocytes (*p* = 2.9E-4), macrophages (*p* = 0.02), M1 macrophages (*p* = 9.9E-3), monocytes (*p* = 0.04), mesenchymal stem cells (MSC, *p* = 0.03), natural killer T cells (NKT, *p* = 0.02), sebocytes (*p* = 5.0E-4), and gamma delta T cells (Tgd, *p* =0.03) and a negative correlation with smooth muscle (*p* = 1.0E-3) (**Figures 3C**). These results suggest that a high E6ratio may contribute to few tumor killer cells (aDC, M1 macrophages, monocytes, NKT and Tgd) infiltration in microenvironment, and ultimately lead to a poorer prognosis in cervical cancer.

### Differentially expressed genes identifications and functional enrichment analysis based on E6 splicing in cervical cancer

**Figure 4:**
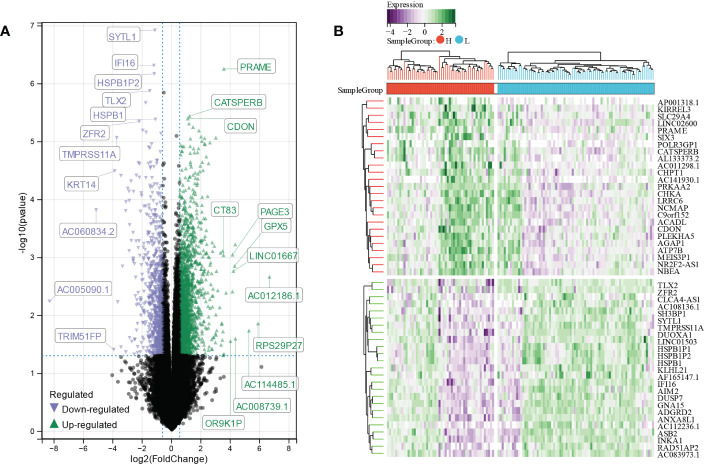
Identification of E6 splicing DEGs in CESC **(A)** The volcano plot of all DEGs in high and low E6ratio TCGA-CESC patients. The differences are set as p value<0.05 and log FCI >1.5 **(B)** Heatmap showing 50 significantly DEGS of high and low E6ratio TCGA-CESC patients.

A total of 2923 differentially expressed genes (DEGs) were screened out by comparing high E6 ratio samples with low E6 ratio samples, including 1971 upregulated and 951 downregulated DEGs (**Figure 4A**). After excluding the genes with *p* ≥ 0.0001, we obtained 118 DEGs, of which 68 were upregulated and 50 were downregulated (**Figure 4B**; **Table S1**).

**Figure 5:**
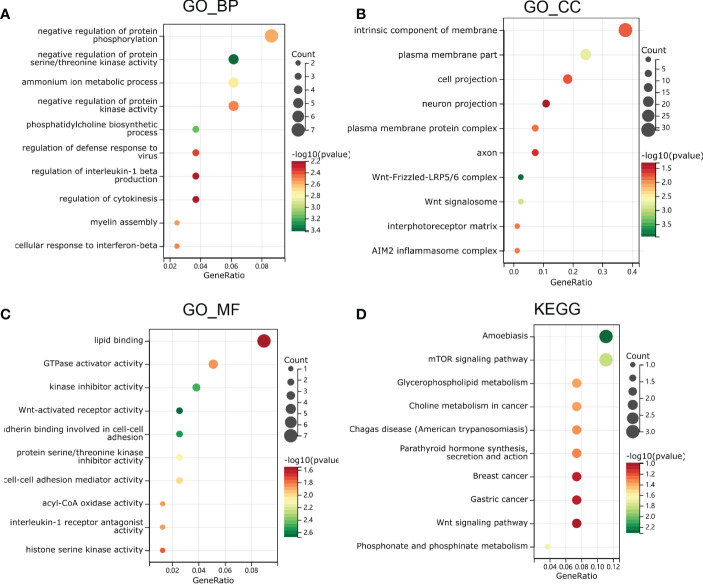
Functional enrichment analysis in GO and KEGG. **(A)** biological process, **(B)** molecular function and **(C)** cell composition of GO enrichment analysis. **(D)** Kyoto Encyclopedia of Genes and Genomes analysis results of differentially expressed genes between high and low E6ratio group. The words on the left indicates enriched terms, the size of the balls indicates the number of the genes enriched and the color indicates the level of the enrichment.

To further investigate the function and mechanism of E6ratio in cervical cancer, we further performed enrichment analysis of DEGs. The upregulated and downregulated 118 DEGs were processed separately for Gene Ontology (GO) and Kyoto Encyclopedia of Genes and Genomes (KEGG) pathway analyses. The top five significantly enriched biological processes (BP) were the negative regulation of protein serine/threonine kinase activity, CDP-choline pathway, phosphatidylcholine biosynthetic process, the positive regulation of interleukin (IL)-1 beta production, and assembly of hemidesmosome (**Figure 5A**). The top five significantly enriched cellular components (CC) were the Wnt-Frizzled-LRP5/6 complex, Wnt signalosome, plasma membrane part, an intrinsic component of the plasma membrane, and an extrinsic component of the plasma membrane (**Figure 5B**). The top five significantly enriched molecular functions (MF) were coreceptor activity involved in the canonical Wnt signaling pathway, coreceptor activity involved in the Wnt signaling pathway, Wnt-activated receptor activity, cadherin binding involved in cell-cell adhesion, and kinase inhibitor activity (**Figure 5C**). The significantly enriched KEGG pathways were amoebiasis, mTOR signaling pathway, phosphonate and phosphinate metabolism, glycerophospholipid metabolism, and choline metabolism in cancer (**Figure 5D**). These findings imply that E6 has a broad influence on signaling, immunological control, and metabolism.

**Figure 6:**
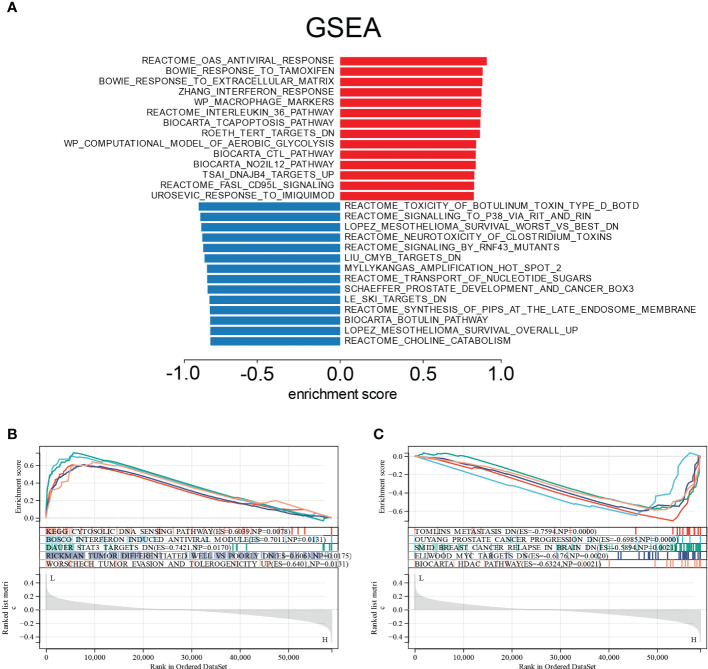
Enrichment plots from GSEA. **(A)** Significantly enriched pathway annotations of CESC. **(B)** Significantly positive enriched pathways in low E6ratio CESC patients. **(C)** Significantly positive enriched pathways in high E6ratio CESC patients.

We used Gene Set Enrichment Analysis (GSEA) on the E6 splicing DEG to find GO and signaling pathways that were differently active in cervical cancer to corroborate these findings. The top 20 significantly positive and negative enrichment pathways are shown in **Figure 6A**. GSEA demonstrated that the significantly positively enriched pathways in the low E6 ratio were cytosolic DNA sensing pathway, interferon-induced antiviral module, STAT3 targets, tumor differentiated, tumor evasion, and tolerogenicity (**Figure 6B**). Gene sets related to metastasis, prostate cancer progression, breast cancer relapse in the brain, MYC targets, and HDAC pathway showed enrichment in the patients with a high E6 ratio (**Figure 6C**).

### Identification of DElncRNA, DEmiRNA, and DEmRNA based on E6 splicing

**Figure 7:**
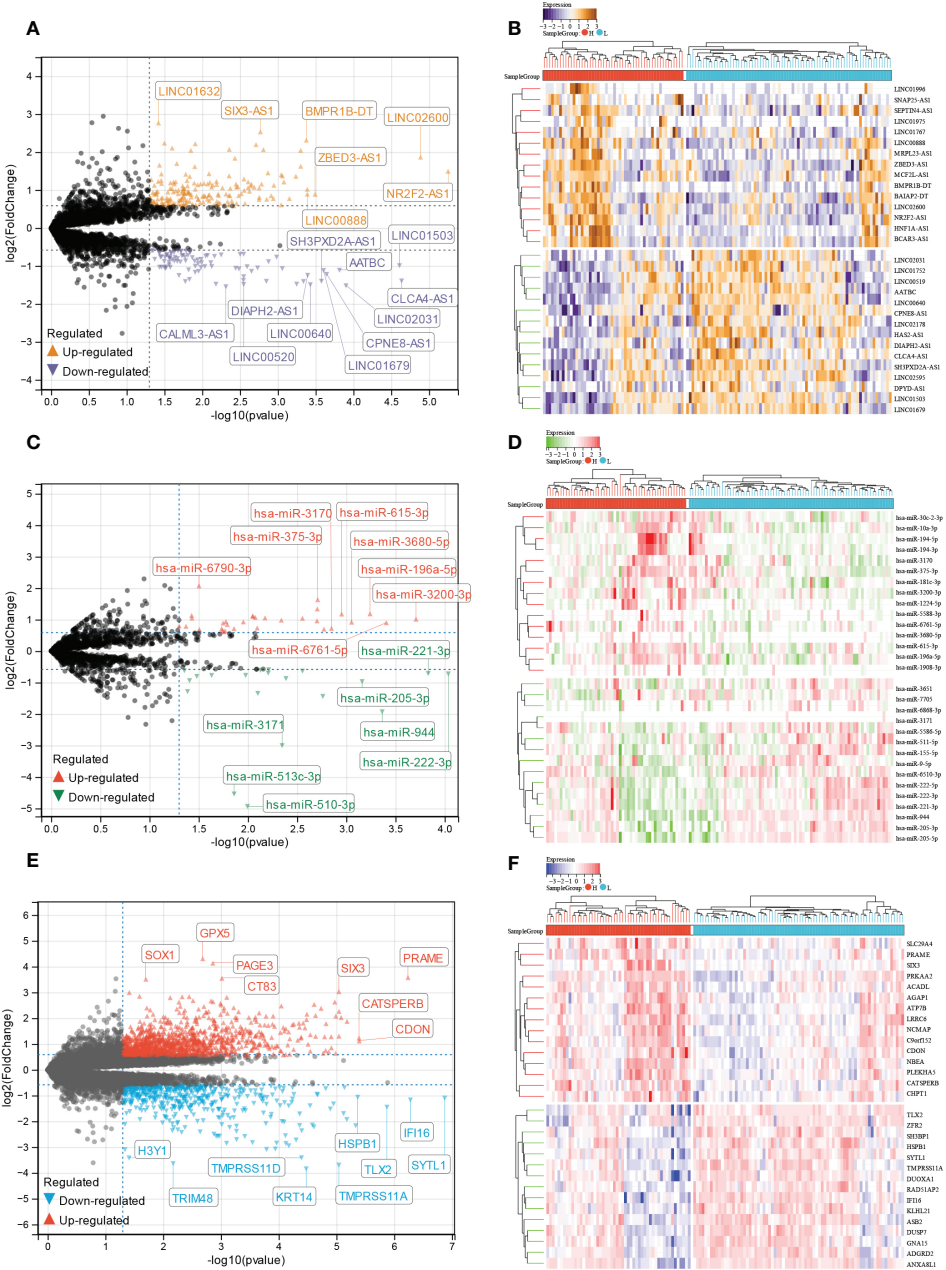
Identification of E6 splicing DEGs in CESC The volcano plot of **(A)** DEIncRNAs **(C)** DEmiRNAs **(E)** DEmRNAs in high and low E6ratio CESC patients. The differences are set as P value<0.05 and |log FC| >1.5. Heatmap showing 30 significantly **(B)** DEIncRNAs **(D)** DEmiRNAs **(F)** DEmRNAs of high and low E6ratio CESC patients.

We analyzed differentially expressed lncRNA (DElncRNA), differentially expressed miRNA (DEmiRNA), and differentially expressed mRNA (DEmRNA) between high and low E6 ratio CESC samples. A total of 277 DElncRNA (187 upregulated and 90 downregulated; **Figure 7A, B**), 101 DEmiRNA (57 upregulated and 44 downregulated; **Figure 7C, D**), and 1617 DEmRNA (1098 upregulated and 519 downregulated; **Figure 7E, F**) were identified as differentially expressed RNA in CESC. All DElncRNA, DEmiRNA, and DEmRNA with their names, log2FC values, and *p*-values are listed in **Supplementary Tables S2**-**4**.

### Construction of immune-related ceRNA network based on E6 splicing

**Figure 8:**
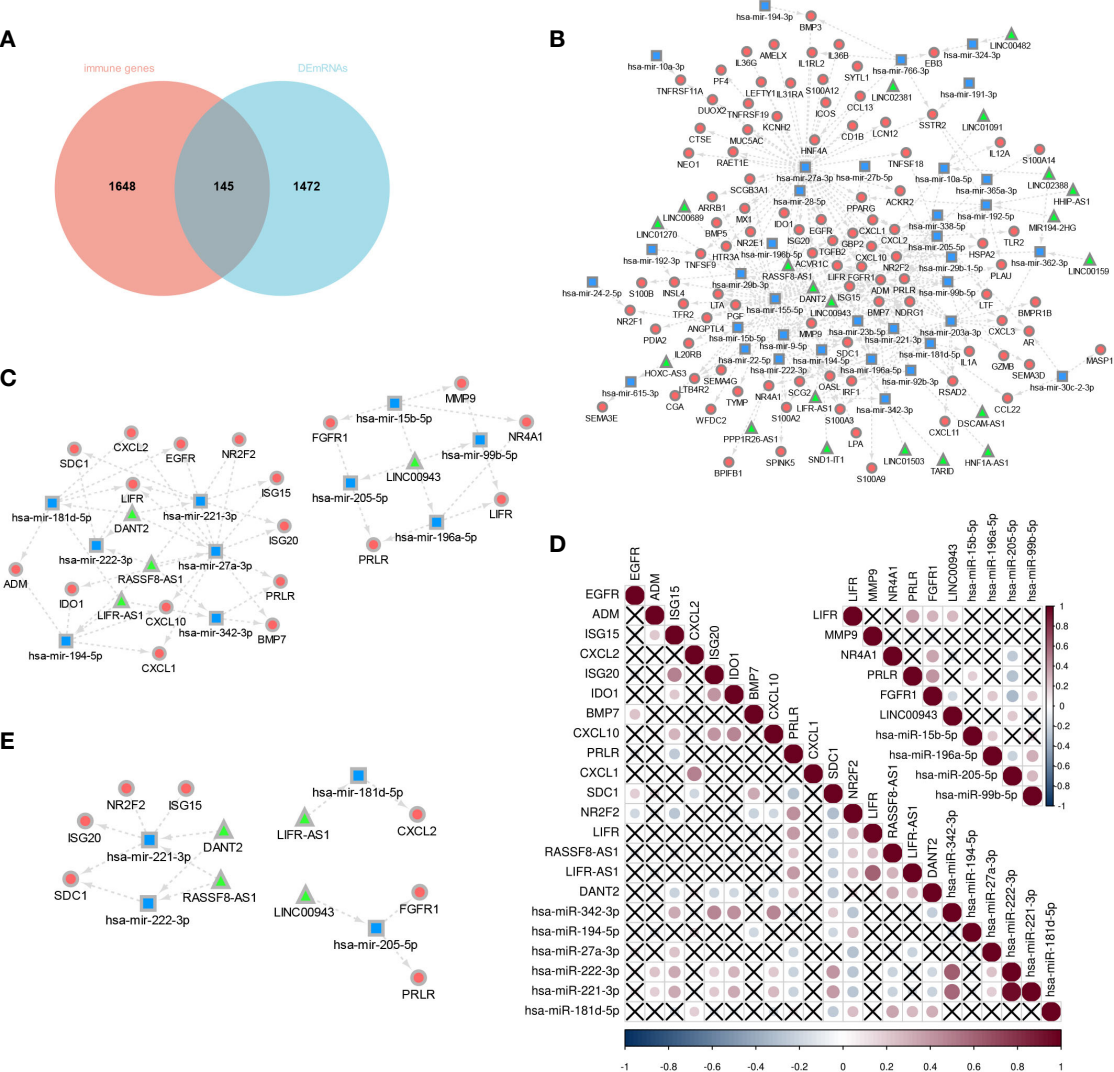
Construction of E6 splicing Immune-related ceRNA Networks. **(A)** Intersection between DEmRNA and immune genes. **(B)** ceRNA network among IncRNAs, miRNAs, and mRNAs, Circular, mRNAs: square, IncRNAs; triangle, miRNAs. **(C)** A subnetwork from the ceRNA network **(D)** Co-expression analysis of DEGS in sub-network. **(E)** 14 hub genes of E6 splicing Immune-related ceRNA sub-network.

The Venny method was used to analyze the intersection between DEmRNA and immune genes (**Figure 8A**), and a total of 146 immune-related DEmRNA were screened.

To construct a ceRNA network among 277 DElncRNA, 101 DEmiRNA, and 146 immune-related DEmRNA, we further predicted lncRNA-miRNA and miRNA-mRNA interactions using the miRNet database. These differentially expressed lncRNA-miRNA-immune gene interaction pairs were then integrated to construct the ceRNA network (**Figure 8B**). Additionally, we extracted a sub-network from the abovementioned ceRNA network, including four lncRNA, 10 miRNA, and 18 mRNA, which comprised two modules (**Figure 8C**). To further validate the network, we aimed to estimate the correlation of these genes in the TCGA-CESC cohort. The co-expression plot among these genes is shown in **Figure 8D**. With the aid of the co-expression plot, we identified 14 hub genes of the immune-related ceRNA sub-network based on E6 splicing, namely three lncRNA (LINC00943, LIFR-AS1, DANT2, and RASSF8-AS1), four miRNA (miR-205-5p, miR-181d-5p, miR-222-3p, and miR-221-3p), and seven mRNA (*FGFR1*, *PRLR*, *CXCL2*, *ISG20*, *ISG15*, *SDC1*, and *NR2F2*) (**Figure 8E**).

### Regulators of immune-related ceRNA network based on E6 splicing in cervical cancer

**Figure 9:**
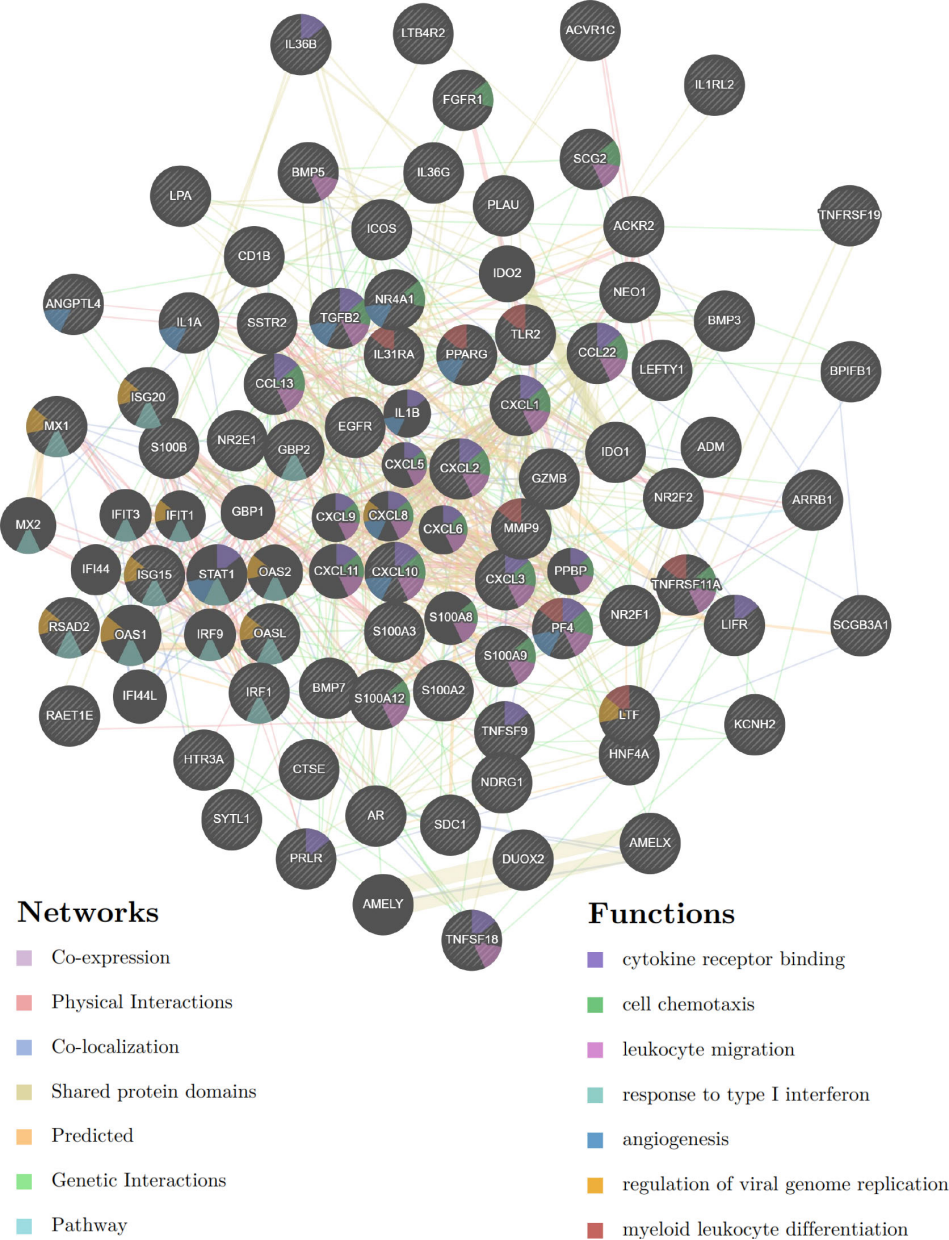
Protein-protein interaction network based on the E6 splicing immune-related ceRNA networks in the TCGA-CESC cohort.

GeneMANIA was used to create a protein-protein interaction network based on the ceRNA network in the TCGA-CESC cohort. We found that these ceRNA network genes play key roles in cytokine receptor binding, cell chemotaxis, leukocyte migration, response to type I interferon, angiogenesis, viral genome replication regulation, and myeloid leukocyte differentiation (**Figure 9**).

**Figure 10:**
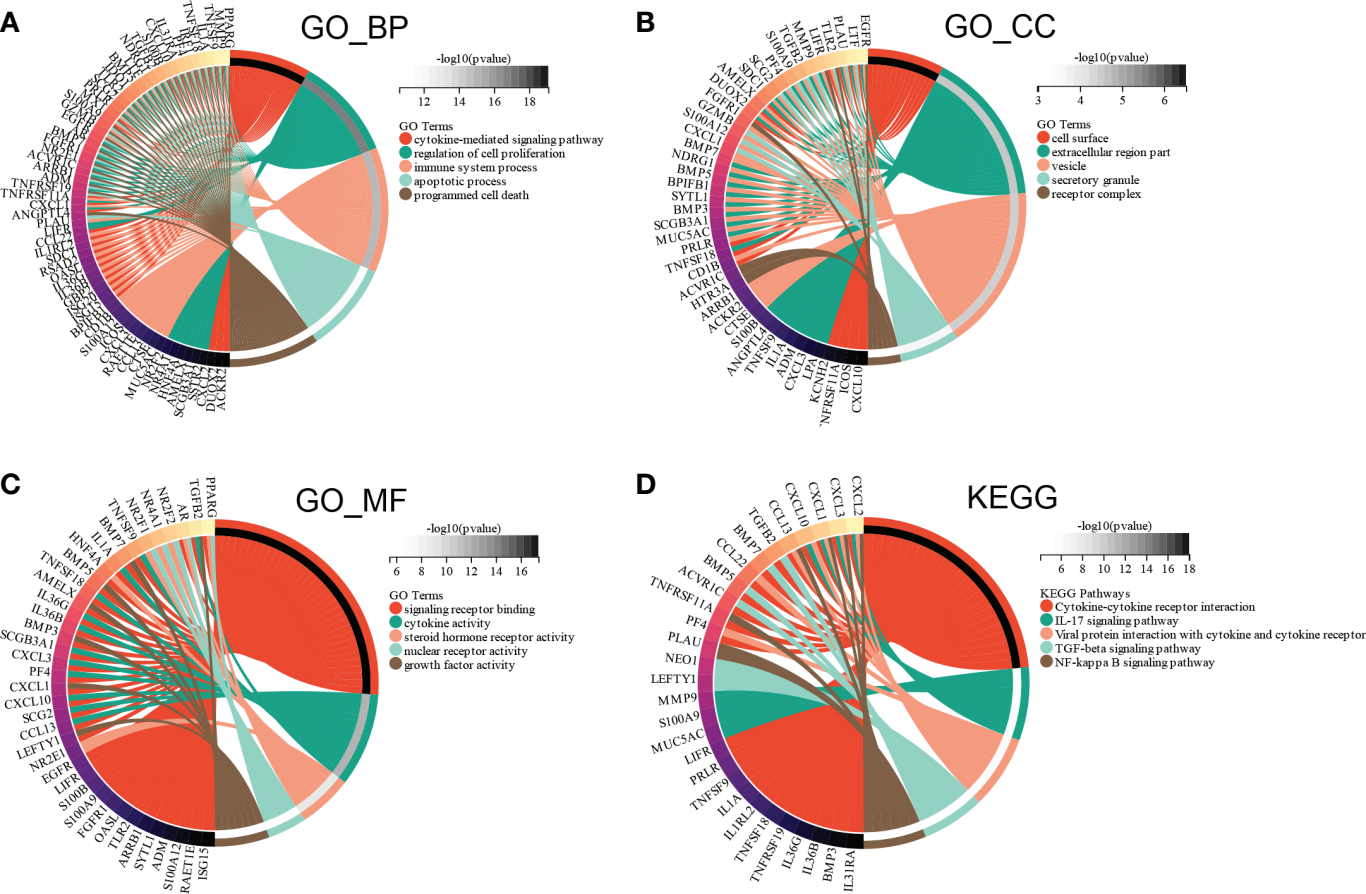
Functional enrichment analysis of ceRNA Network. **(A)** biological process, **(B)** molecular function **(C)** cell composition and **(D)** KEGG analysis results of differentially expressed immune-related genes between high and low E6ratio group.

Subsequently, GO and KEGG enrichment analyses were conducted to investigate the functions of the ceRNA network. BP analysis showed that the ceRNA network was significantly enriched in the cytokine-mediated signaling pathway, regulation of cell proliferation, immune system process, apoptotic process, and programmed cell death (**Figure 10A**). CC analysis indicated that the ceRNA network was significantly enriched in cell surfaces, extracellular regions, vesicles, secretory granules, and receptor complexes (**Figure 10B**). MF analysis showed that it was significantly enriched in signaling receptor binding, cytokine activity, steroid hormone receptor activity, nuclear receptor activity, and growth factor activity (**Figure 10C**). The KEGG pathway enrichment analysis showed that the ceRNA network was significantly enriched in cytokine-cytokine receptor interaction, IL-17 signaling pathway, viral protein interaction with cytokine and cytokine receptor, TGF-beta signaling pathway, and NF-kappa B signaling pathway (**Figure 10D**).

### Immune-related ceRNA network based on E6 splicing is associated with survival and immune cell infiltration in cervical cancer

**Figure 11:**
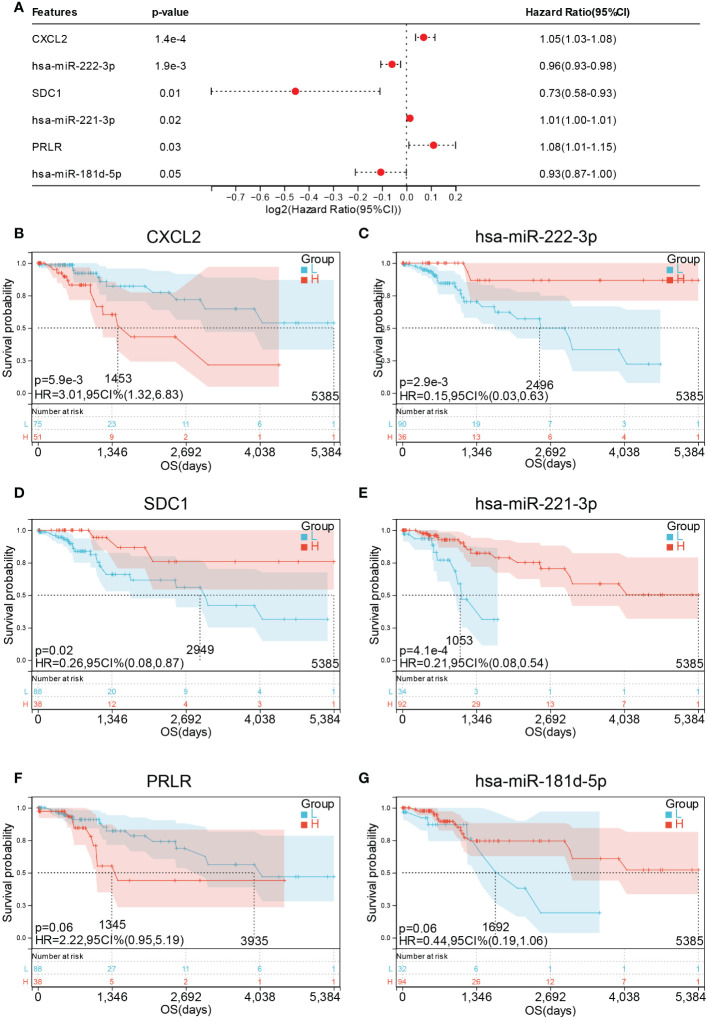
Prognostic values of hub genes in CESC patients. **(A)** forest plot of 6 hub genes in TCGA-CESC cohort. Prognostic value of **(B)** CXCL2 **(C)** miR-222-3p **(D)** SDC1 **(E)** miR-221- 3P **(F)** PRLR and **(G)** miR-181d-5p in TCGA-CESC cohort.

To further confirm the relationship between the 14 hub genes and the prognosis of CESC patients, OS and expression levels of these hub genes were detected using forest plot. Only six genes were found to be significantly associated with prognosis (**Figure 11A**). Survival analysis results suggest that high expression levels of *CXCL2* (HR = 3.01, *p* = 5.9E-3) was associated with shorter OS in CESC (**Figure 11B**), whereas high expression levels of has-miR-222-3p (HR = 0.15, *p* = 2.9E-3), *SDC1* (HR = 0.26, *p* = 0.02), and has-miR-221-3p (HR = 0.21, *p* = 4.1E-4) were associated with longer OS in CESC (**Figure 11C–E**). Meanwhile, *PRLR* (HR = 2.22, *p* = 0.06) and has-miR-181d-3p (HR = 0.44, *p* = 0.06) exhibited no significant association with OS (**Figure 11F, G**).

**Figure 12:**
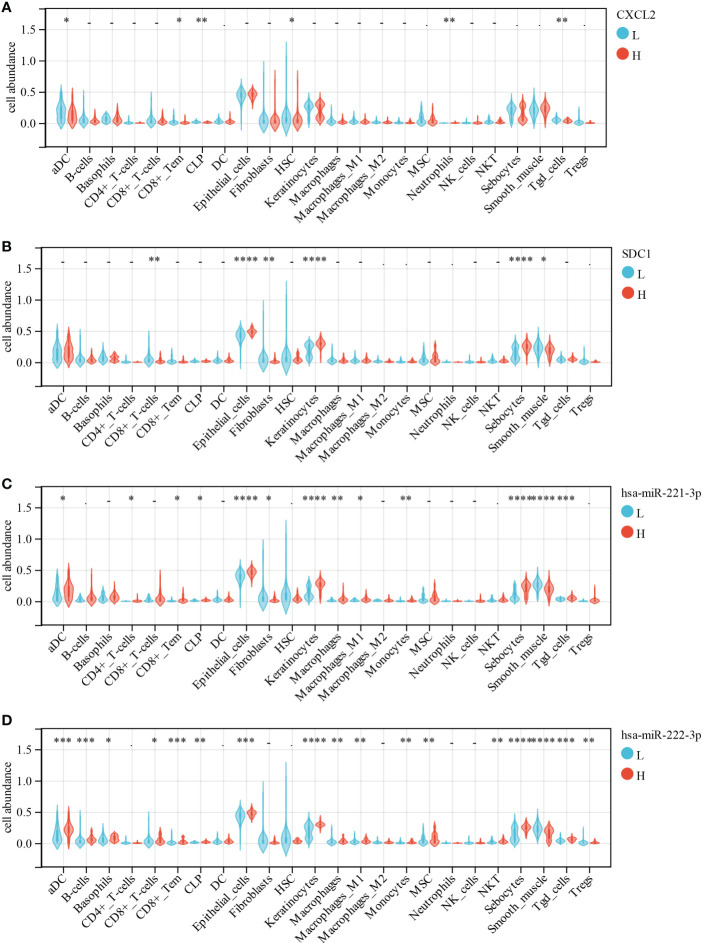
Correlations of low/high **(A)** CXCL2, **(B)** SDC1, **(C)** miR-221-3p and **(D)** miR-222-3p expression with immune cells infiltration in CESC. *, *p* < 0.05; **, *p* < 0.01; ***, *p* < 0.001.

Furthermore, the correlation between immune cell abundance and DEG was analyzed using Wilcoxon test. *CXCL2* expression had a significantly negative correlation with aDC (*p* = 0.02), CD8+ effector memory T cell (Tem, *p* = 0.002), CLP (*p* = 7.80E-03), neutrophils (*p* = 7.50E-03), and Tgd (*p* = 4.00E-03; **Figure 12A**). *SDC1* expression displayed a significantly positive correlation with epithelial cells (*p* = 4.50E-07), keratinocytes (*p* = 1.70E-08), and sebocytes (*p* = 2.10E-07) but a significantly negative correlation with CD8+ T cells (*p* = 8.50E-03), fibroblasts (*p* = 8.90E-03), and smooth muscle (*p* = 0.02; **Figure 12B**). We found that miR-221-3p expression had a significantly positive correlation with aDC (*p* = 0.03), CD4+ T cells (p = 0.01), CD8+ Tem (*p* = 0.01), CLP (*p* = 0.02), epithelial cells (*p* = 2.60E-06), keratinocytes (*p* = 5.70E-09), macrophages (*p* = 5.50E-03), M1 macrophages (*p* = 0.01), monocytes (*p* = 2.90E-03), sebocytes (*p* = 6.70E-08), and Tgd (*p* = 5.10E-04; **Figure 12C**). In contrast, it was significantly negatively correlated with fibroblasts (*p* = 0.04) and smooth muscle (*p* = 6.60E-06; **Figure 12C**). Moreover, miR-222-3p expression had a significantly positive correlation with aDC (*p* = 1.20E-04), B cells (*p* = 1.10E-04), basophils (*p* = 0.01), CD8+ T cells (*p* = 0.05), CD8+ Tem (*p* = 2.60E-04), CLP (*p* = 5.40E-03), epithelial cells (*p* = 5.40E-04), keratinocytes (*p* = 1.60E-08), macrophages (*p* = 2.30E-03), M1 macrophages (*p* = 1.10E-03), monocytes (*p* = 6.20E-03), MSC (*p* = 9.70E-03), NKT (*p* = 8.70E-03), sebocytes (*p* = 6.40E-07), and Tgd (*p* = 1.50E-04; **Figure 12D**). However, it was significantly negatively correlated with smooth muscle (*p* = 6.60E-05) and regulatory T cell (Treg, *p* = 6.30E-03; **Figure 12D**).

### High E6 ratio enhances the proliferation and migration of cervical cancer cells

**Figure 13:**
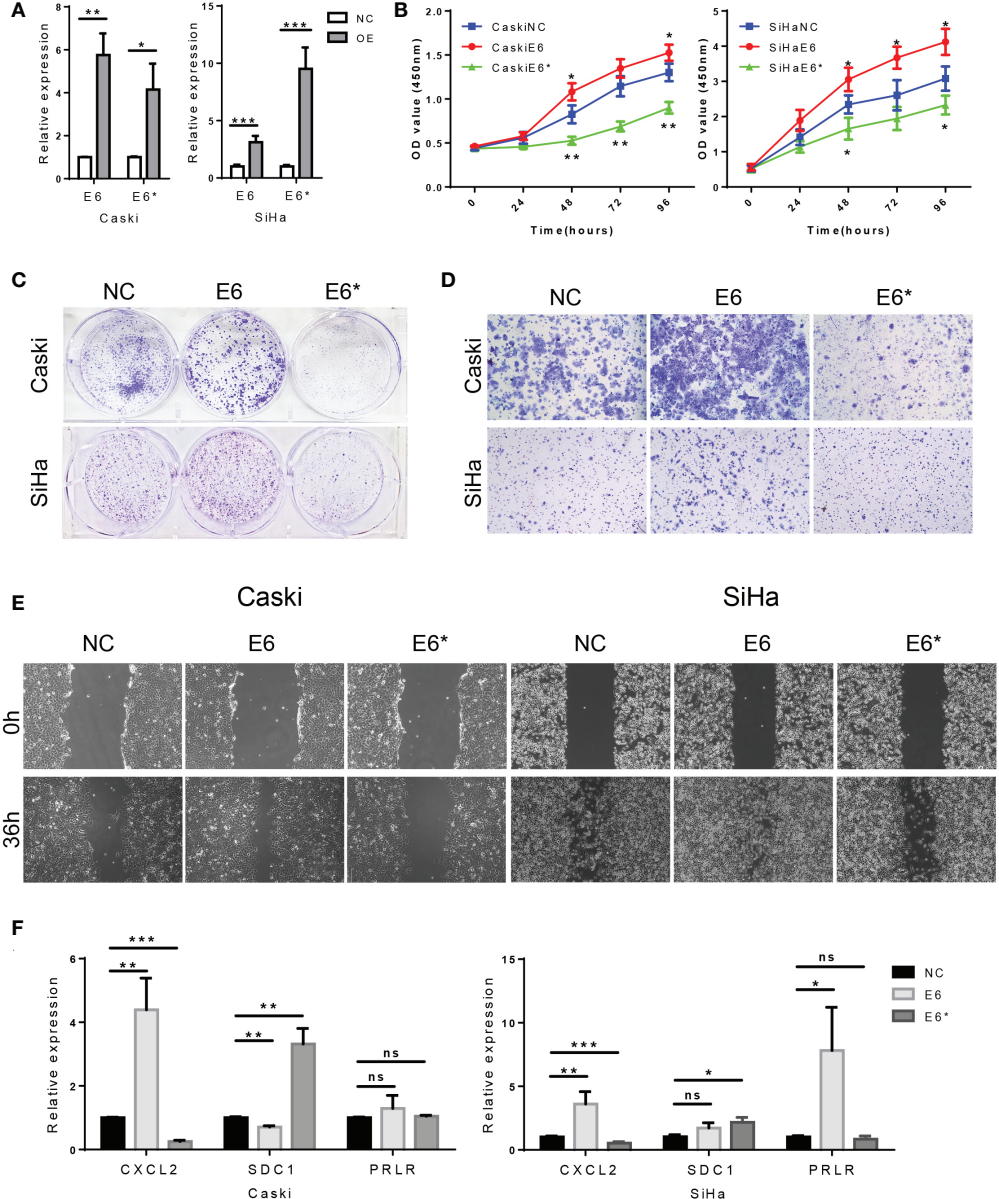
High E6/E6* ratio enhances proliferation and migration of cervical cancer cells in vitro. **(A)**. RT-PCR demonstrate overexpression of E6 and E6* in Caski cells and SiHa cells. **(B, C)**. The proliferation was examined by CCK-8 and colony-forming assays. **(D, E)**. The migration and invasion were examined by wound healing and transwell assays. **(F)**. Related DEGs' RNA expression was analysed using RT-PCR. ns, not significant; *, p < 0.05; **, p < 0.01; ***, p < 0.001.

Based on the above results, we hypothesized that E6/E6* ratio may affect the malignant behavior of cervical cancer cells. Using a plasmid transfection system, we changed the E6ratio of SiHa and Caski by overexpressing E6 or E6*. The levels of E6 and E6* in these resultant cell lines were then determined by RT-PCR (**Figure 13A**).

CCK-8 assays indicated that the cell proliferation rate was increased by E6 overexpression (E6-OE), while E6* overexpression (E6*-OE) inhibits cell proliferation in SiHa and Caski cells (**Figure 13B**). In addition, the same conclusion was obtained in the colony forming experiment (**Figure 13C**). These results suggest that high E6 ratio promotes cell proliferation in cervical cancer cells.

Transwell assays showed, however, that E6-OE cervical cancer cells exhibited a higher migration capacity than vector control cells, whereas E6*-OE cells had a lower migration capacity (**Figure 13D**). Wound healing assays indicated that the cell migratory speed was changed by E6 (**Figure 13E**); These results suggest that high E6 ratio promotes migration capacity in cervical cancer cells.

Moreover, we tested the relative expression levels of CXCL2, SDC1 and PRLR by RT-PCR, which demonstrated that the expression levels of CXCL2 were increased in the group of E6-OE, while CXCL2 expression were decreased in the group of E6*-OE. Moreover, the expression levels of SDC1 were increased in the group of E6*-OE. However, we found that the expression of PRLR was not significantly correlated with E6/E6* expression (**Figures 13F**). These results suggested that E6 ratio are critical for immune regulation in these tested cells’ system.

## Discussion

The viral E6 proteins are essential for HPV-positive cancer cells to maintain their malignant character. Several studies have found that HPV-positive cancer cells are “oncogene addicted,” meaning that their proliferation is reliant on E6 expression (29). In cervical cancer, E6 oncoproteins have been demonstrated to maintain proliferative signaling, elude growth suppressors, stimulate tissue invasion and metastasis, permit replicative immortality, promote angiogenesis, and resist cell death (30). The splicing process is a critical regulator of gene expression and a contributor to cell proteome diversity. Mechanisms of alternative splicing include RNA-protein interactions of splicing factors, RNA-RNA base-pairing interactions, or chromatin-based effects (31). Various splicing factors have been identified to impact the production of E6 splicing variants. Overexpression of hnRNP A1 causes a rise in unspliced E6 mRNAs, whereas overexpression of hnRNP A2 causes E6 alternative splicing (32). Rosenberger et al. found that EGF suppresses spliced E6 mRNA production while boosting unspliced E6 mRNA production (33). SRSF1 acts as a splicing enhancer, increasing the expression of E6/E7 (34). CTCF mutation increases the synthesis of unspliced E6 and spliced E6*I (35). Mirza S et al. reported that knockdown ECD lead to aberrations in E6/E7 RNA splicing, as well as RNA splicing of several HPV oncogenesis-linked cellular genes (36).

The thoroughly studied transcripts for HPV16 are E6*I, E6*II, E6*III, E6^E7, E6^E7*I, E6^E7*II, E6*IV, E6*V, and E6*VI, and the transcripts for HPV18 include E6*I, E6*II, E6*III, and E6^E7 (9). E6* expression has been linked to anti-tumorigenic characteristics in previous studies. E6*I and E6*III transcripts were found in cervical cancer, as well as low and high-grade lesions, where no E6 mRNA was found (37). By binding to E6AP, E6, and p53, the E6*I protein prevents p53 degrading (9). Additionally, Overexpression E6* alters β-integrin and mitochondrial dysfunction pathways in cervical cancer (38). Another research found that E6*I promotes the overexpression of E-cadherin protein and suppresses the malignancy of tumor cells (39). In our study, the E6 ratio (E6/E6*) was significantly negatively correlated with OS, PFS, and HPV16 infection in cervical cancer. However, other clinical variables such as age, height, weight, BMI, histological type, stage, and grade in cervical cancer were not shown to be substantially linked with the E6 ratio. Our research also imply that E6 has a broad influence on cytosolic DNA sensing pathway, interferon-induced antiviral module, STAT3 targets, tumor differentiated, tumor evasion, and tolerogenicity. Targeting E6 splicing for functional inactivation might be a useful therapeutic technique since it plays a vital role in cervical cancer cells. The SF3B1 inhibitor meayamycin B reduces the amount of the transcript E6*I while increasing the number of unspliced E6 mRNAs (40). However, the efficacy of E6 splicing inhibitors still needs to be verified by more basic and clinical experiments.

CeRNA has emerged as a possible class of post-transcriptional regulators that regulate gene expression *via* a miRNA-mediated mechanism in recent years (41). The ceRNA network has been confirmed to be involved in various cancers, including cervical cancer. In our study, we identified 14 hub genes in the immune-related ceRNA sub-network based on E6 splicing, namely three lncRNA (*LINC00943, LIFR-AS1, DANT2*, and *RASSF8-AS1*), four miRNA (miR-205-5p, miR-181d-5p, miR-222-3p, miR-221-3p), and seven mRNA (*FGFR1*, *PRLR*, *CXCL2*, *ISG20*, *ISG15*, *SDC1*, and *NR2F2*). Furthermore, only four genes (*CXCL2*, *SDC1*, has-miR-221-3p, and has-miR-222-3p) were associated with OS in cervical cancer. CXCL2 is generated by activated monocytes and neutrophils and expressed at inflammatory areas; it inhibits the growth of hematopoietic progenitor cells (42). According to Wan et al., AKIP1 has a positive correlation with CXCL1/CXCL2 and is related with advanced tumor characteristics and poor survival profiles in cervical cancer patients (43). CXCL2 is associated with lymph node metastasis in cervical cancer (44). Syndecans are plasma membrane proteoglycans that are considered to interact with the actin cytoskeleton through their cytoplasmic domain. In cervical cancer, the presence of Syndecan-1 (SDC1) in the cytoplasm of tumor cells predicts improved patient survival (45). Furthermore, tumor cell growth can be aided by SDC1-positive fibroblasts *in vitro* (46). Wang et al. indicated that has-miR-221-3p/has-miR-222-3p and target genes, particularly *CBFB* and *UBE2N*, may serve as prognostic indicators for hepatocellular carcinoma (47). Wei et al. discovered that miR-221-3p has a mechanistic role in lymph node metastasis, implying that miR-221-3p is increased by the transcription factor TWIST2 and downregulates its target THBS2, potentially promoting lymph node metastasis in cervical cancer (48). miR−222−3p is a canonical factor that regulates the expression and signaling of many genes involved in tumor initiation and progression (49). The findings of Fan et al. suggest that SNAI2’s non-canonical signaling pathway causes EMT in ovarian cancer cells by decreasing miR-222-3p transcription and upregulating PDCD10 expression (50). Ying et al. demonstrated that exosomal miR-222-3p released by epithelial ovarian cancer causes the polarization of tumor-associated macrophages (51).

Immunotherapy is a potential treatment option for cervical cancer, especially because it targets non-self-viral antigens (6). Due to the ongoing activation of these oncogenes throughout disease development, E6- and E7-specific cellular and humoral immune responses have been somewhat effective (52). By stimulating the immune system, a new plasmid containing synthetic E6 and E7 DNA sequences resulted in histopathological regression and HPV elimination (53). Targeting E6 genetically altered T cells can lead to the regression of HPV16-infected cervical malignancies, according to Draper et al. (54). Immunotherapy treating HPV-positive malignancies, on the other hand, has had mixed results thus far. This might be due to the fact that oncogenic HPV can cause a variety of immune evasion strategies, including antigen processing and presentation dysfunction and cytokine signaling subversion (29).

Antibodies that target PD-1/PD-L1 and CTLA4 are a well-studied and widely used method in cancer immunotherapy (55). Multiple trials have recently explored the potential advantages of immune checkpoint inhibitors (ICI), and pembrolizumab was recently approved by the US FDA for chemotherapeutically treating patients with recurrent cervical cancer and progression, based on a response rate of 14% (8). HPV-related cervical malignancies and intraepithelial neoplasia have been shown in studies to upregulate PD-L1, which is another significant target molecule for ICI treatment (56). Although ICI immunotherapy appears promising, the poor response rate is a major concern. Several studies have discovered that the tumor microenvironment is mostly responsible for immunotherapy’s ineffectiveness or poor response (TME) (57). TME immune cells, such as Treg, tumor-associated macrophages, and myeloid-derived suppressor cells, might be a potential technique for improving ICI response rates (58).

Rather of focusing primarily on antigen-specific T cells, a wider, more successful strategy might include addressing immunosuppression in the TME, strengthening effector immune cells, or combining various techniques (6). A booster immune response might be induced by combining antigen-specific therapeutic vaccines with antibodies to reverse immunosuppression and promote effector T cells (8). Our results show that E6 splicing was not only associated with clinical features and survival, but also correlated with the low infiltration of effector immune cells, such as aDC, M1 macrophages, monocytes, NKT, and Tgd, in cervical cancer. Moreover, our analysis showed that the ceRNA network was significantly enriched in the cytokine-mediated signaling pathway, immune system process, and programmed cell death. Our findings not only shed light on the processes governing E6 splicing and tumor-immune interactions in cervical cancer, but also on the theoretical underpinnings of combinatorial methods.

## Conclusion

In this comprehensive study, we found that E6 splicing was correlated with the survival of patients with cervical cancer. The high E6 ratio had a significantly negative correlation with the infiltrating levels of inflammatory cells, such as aDC, M1 macrophages, monocytes, NKT, and Tgd, in cervical cancer. E6 splicing exhibited a widespread impact on the functional enrichment analysis, indicating that high E6 ratio phenotypes were largely involved in immune response regulation, cell adhesion, and Wnt signaling pathways. Subsequently, we constructed an immune-related ceRNA network based on E6 splicing, including 277 lncRNA, 101 miRNA, and 146 mRNA. This ceRNA network was found to regulate several immune-related signaling pathways in cervical cancer. Furthermore, we identified a ceRNA sub-network including three lncRNA (*LINC00943, LIFR-AS1, DANT2*, and *RASSF8-AS1*), four miRNA (miR-205-5p, miR-181d-5p, miR-222-3p, and miR-221-3p), and seven mRNA (*FGFR1*, *PRLR*, *CXCL2*, *ISG20*, *ISG15*, *SDC1*, and *NR2F2*). Among them, *CXCL2*, *SDC1*, and has-miR-221-3p were considered to be important DEG associated with survival and immune cell infiltration. Taken together, our findings suggest that the ceRNA network based on E6 splicing plays a crucial role in immune response regulation in cervical cancer; nonetheless, the underlying mechanisms still need further validation.

## Data availability statement

The original contributions presented in the study are included in the article/**Supplementary Materials**. Further inquiries can be directed to the corresponding author.

## Ethics statement

Ethical review and approval was not required for the study on human participants in accordance with the local legislation and institutional requirements. Written informed consent for participation was not required for this study in accordance with the national legislation and the institutional requirements.

## Author contributions

Conceptualization: SJ and YZ. Supervision: YY. Data curation: SJ and MY. Methodology: YY and YZ. Visualization: SJ. Vitro experiment: SJ. Original draft: SJ. Review & editing, YZ and LZ. Funding acquisition: XL. All authors contributed to the article and approved the submitted version.
