# Supplementary material for: Construction of an immune-related ceRNA network in cervical cancer based on HPV E6 splicing
Source: Front Oncol. 2022 Dec 14;12:979884. doi: 10.3389/fonc.2022.979884 (PMC9796994; doi:10.3389/fonc.2022.979884)
Supplement: Supplementary file 1 [file DataSheet_1.docx]

| Table S1. E6 splicing Significantly Correlated Genes (*p* <0.0001). | | | | |
| --- | --- | --- | --- | --- |
| Tag | logFC | t | *p*-Value | adjust.*p*-Value |
| SYTL1 | -1.09989 | -5.60499 | 1.21E-07 | 0.006084 |
| IFI16 | -1.17405 | -5.29758 | 4.93E-07 | 0.008605 |
| PRAME | 3.590751 | 5.294893 | 5.61E-07 | 0.008605 |
| HSPB1P2 | -1.14712 | -5.22404 | 6.85E-07 | 0.008605 |
| TLX2 | -1.45365 | -5.07217 | 1.35E-06 | 0.012098 |
| AC112236.1 | -1.73423 | -5.01239 | 2.17E-06 | 0.015589 |
| CATSPERB | 1.244093 | 4.839071 | 3.67E-06 | 0.018557 |
| CDON | 1.132249 | 4.818248 | 4.03E-06 | 0.018557 |
| HSPB1 | -1.067 | -4.80766 | 4.19E-06 | 0.018557 |
| ZFR2 | -2.17356 | -4.79089 | 4.56E-06 | 0.018557 |
| POLR3GP1 | 1.21548 | 4.901063 | 4.92E-06 | 0.018557 |
| NR2F2-AS1 | 1.493185 | 4.740054 | 5.65E-06 | 0.018557 |
| NBEA | 1.892037 | 4.703987 | 6.48E-06 | 0.018557 |
| ADGRD2 | -1.9327 | -4.71175 | 6.49E-06 | 0.018557 |
| RAD51AP2 | -1.70324 | -4.71656 | 6.98E-06 | 0.018557 |
| SH3BP1 | -0.69559 | -4.67345 | 7.36E-06 | 0.018557 |
| PRKAA2 | 2.040346 | 4.647087 | 8.26E-06 | 0.018557 |
| SIX3 | 3.04795 | 4.696289 | 8.66E-06 | 0.018557 |
| TMPRSS11A | -3.70222 | -4.64856 | 8.67E-06 | 0.018557 |
| ACADL | 2.278355 | 4.649442 | 8.83E-06 | 0.018557 |
| SLC29A4 | 1.317016 | 4.625984 | 8.96E-06 | 0.018557 |
| NCMAP | 2.098598 | 4.60247 | 9.88E-06 | 0.018557 |
| C9orf152 | 2.642808 | 4.592504 | 1.05E-05 | 0.018557 |
| AC141930.1 | 2.266156 | 4.685824 | 1.1E-05 | 0.018557 |
| ASB2 | -1.32836 | -4.57401 | 1.11E-05 | 0.018557 |
| AC011298.1 | 2.312978 | 4.611677 | 1.12E-05 | 0.018557 |
| PLEKHA5 | 0.6517 | 4.563571 | 1.16E-05 | 0.018557 |
| LRRC6 | 1.296563 | 4.552876 | 1.21E-05 | 0.018557 |
| CHPT1 | 0.866182 | 4.551841 | 1.22E-05 | 0.018557 |
| AP001318.1 | 0.760988 | 4.721169 | 1.22E-05 | 0.018557 |
| DUOXA1 | -1.52466 | -4.54261 | 1.26E-05 | 0.018557 |
| LINC02600 | 1.864633 | 4.533097 | 1.32E-05 | 0.018557 |
| AC083973.1 | -1.44728 | -4.53499 | 1.33E-05 | 0.018557 |
| AL133373.2 | 1.532234 | 4.565446 | 1.33E-05 | 0.018557 |
| CHKA | 0.76302 | 4.504743 | 1.47E-05 | 0.019048 |
| AGAP1 | 0.708845 | 4.504088 | 1.48E-05 | 0.019048 |
| KLHL21 | -0.73604 | -4.49603 | 1.53E-05 | 0.019048 |
| ATP7B | 1.804415 | 4.493315 | 1.54E-05 | 0.019048 |
| MEIS3P1 | 1.591009 | 4.500464 | 1.56E-05 | 0.019048 |
| KIRREL3 | 1.294914 | 4.487005 | 1.59E-05 | 0.019048 |
| EYS | 0.656692 | 4.472266 | 1.69E-05 | 0.019749 |
| CLIC6 | 2.396008 | 4.451692 | 1.83E-05 | 0.020857 |
| DUSP7 | -0.97967 | -4.41195 | 2.14E-05 | 0.022322 |
| GNA15 | -0.95102 | -4.41065 | 2.15E-05 | 0.022322 |
| ASCL5 | 1.312321 | 4.408851 | 2.27E-05 | 0.022322 |
| DRD2 | 2.120555 | 4.412324 | 2.28E-05 | 0.022322 |
| CLCA4-AS1 | -1.38476 | -4.43318 | 2.31E-05 | 0.022322 |
| ANXA8L1 | -2.22043 | -4.38863 | 2.35E-05 | 0.022322 |
| CDH7 | 2.548759 | 4.55258 | 2.4E-05 | 0.022322 |
| LINC01503 | -0.98219 | -4.38183 | 2.42E-05 | 0.022322 |
| AF165147.1 | -1.54105 | -4.38716 | 2.43E-05 | 0.022322 |
| AIM2 | -2.14671 | -4.37667 | 2.47E-05 | 0.022322 |
| AP001094.2 | 1.321074 | 4.414486 | 2.49E-05 | 0.022322 |
| CTAGE3P | 1.984359 | 4.435541 | 2.65E-05 | 0.02247 |
| KCNQ3 | 1.373058 | 4.350893 | 2.73E-05 | 0.02247 |
| INKA1 | -1.39952 | -4.34836 | 2.76E-05 | 0.02247 |
| HSPB1P1 | -1.17182 | -4.34741 | 2.77E-05 | 0.02247 |
| AC108136.1 | -1.4142 | -4.36309 | 2.91E-05 | 0.023042 |
| DENND5B | 1.003028 | 4.333081 | 2.94E-05 | 0.023042 |
| OSBPL10 | 0.733985 | 4.318162 | 3.11E-05 | 0.023979 |
| STXBP6 | 2.170959 | 4.317731 | 3.15E-05 | 0.023979 |
| KRT14 | -3.84644 | -4.30708 | 3.25E-05 | 0.024395 |
| CLDN9 | 1.95809 | 4.304039 | 3.31E-05 | 0.024444 |
| CNKSR2 | 1.638192 | 4.292176 | 3.48E-05 | 0.025365 |
| PKDCC | 1.826878 | 4.279969 | 3.62E-05 | 0.025981 |
| AP000757.1 | 1.494194 | 4.288418 | 3.79E-05 | 0.026831 |
| VSNL1 | -2.36975 | -4.26216 | 3.88E-05 | 0.026911 |
| TMPRSS11D | -3.41685 | -4.26068 | 3.92E-05 | 0.026911 |
| SOBP | 1.281258 | 4.25339 | 4.02E-05 | 0.026911 |
| CENPV | 1.244742 | 4.245407 | 4.15E-05 | 0.026911 |
| AL096870.1 | 0.967749 | 4.342829 | 4.2E-05 | 0.026911 |
| ANXA8 | -2.16364 | -4.24155 | 4.21E-05 | 0.026911 |
| AC211486.1 | -1.30566 | -4.25605 | 4.27E-05 | 0.026911 |
| S100A2 | -1.71533 | -4.23748 | 4.28E-05 | 0.026911 |
| SGSM1 | 1.665553 | 4.238083 | 4.29E-05 | 0.026911 |
| PNMA2 | 1.823777 | 4.233184 | 4.41E-05 | 0.027091 |
| AC008915.3 | 0.606371 | 4.223798 | 4.51E-05 | 0.027299 |
| TMEM150C | 1.162427 | 4.216169 | 4.65E-05 | 0.027788 |
| KRT5 | -2.72674 | -4.20344 | 4.88E-05 | 0.028854 |
| TPO | 1.790979 | 4.2013 | 5.27E-05 | 0.030173 |
| AC009407.1 | 1.619862 | 4.233325 | 5.29E-05 | 0.030173 |
| NRIP3 | -0.95838 | -4.17298 | 5.49E-05 | 0.030698 |
| SAMD9 | -1.13925 | -4.1727 | 5.5E-05 | 0.030698 |
| BTBD3 | 0.800188 | 4.165982 | 5.64E-05 | 0.031038 |
| ARHGEF38 | 1.757783 | 4.163467 | 5.7E-05 | 0.031038 |
| AC082651.3 | -1.44426 | -4.15908 | 5.8E-05 | 0.031038 |
| CDC42EP1 | -0.69788 | -4.15508 | 5.89E-05 | 0.031129 |
| ERVMER34-1 | -1.27064 | -4.14738 | 6.06E-05 | 0.031732 |
| SEMA4G | 1.214333 | 4.136826 | 6.31E-05 | 0.032489 |
| CLEC2B | -1.40277 | -4.13589 | 6.34E-05 | 0.032489 |
| AC007318.1 | -0.80669 | -4.13079 | 6.46E-05 | 0.032762 |
| SPRR1A | -2.95094 | -4.13292 | 6.52E-05 | 0.032762 |
| SCUBE3 | 1.480545 | 4.118189 | 6.78E-05 | 0.033739 |
| FIGNL2 | 0.974538 | 4.123058 | 6.93E-05 | 0.033796 |
| LRP5 | 0.614067 | 4.112628 | 6.93E-05 | 0.033796 |
| AL096828.1 | 0.83087 | 4.221631 | 7.29E-05 | 0.034645 |
| COL25A1 | 1.606063 | 4.116911 | 7.3E-05 | 0.034645 |
| GBP6 | -2.86337 | -4.09969 | 7.31E-05 | 0.034645 |
| CAPNS2 | -2.5609 | -4.09841 | 7.41E-05 | 0.034779 |
| SYNGR3 | -1.27803 | -4.08433 | 7.72E-05 | 0.035885 |
| IL20RB | -2.03121 | -4.08049 | 7.83E-05 | 0.035885 |
| AC099518.6 | -0.91959 | -4.07946 | 7.86E-05 | 0.035885 |
| PPM1H | 1.51203 | 4.07731 | 7.93E-05 | 0.035885 |
| RIN1 | -0.72079 | -4.07485 | 8E-05 | 0.035899 |
| GALNTL6 | 1.658426 | 4.075505 | 8.15E-05 | 0.036142 |
| LRP6 | 0.602509 | 4.068418 | 8.2E-05 | 0.036142 |
| DDX60 | -0.84589 | -4.06372 | 8.35E-05 | 0.036472 |
| SLC10A6 | -2.00741 | -4.05389 | 8.7E-05 | 0.03722 |
| SPDEF | 2.490148 | 4.047951 | 8.86E-05 | 0.03722 |
| TRAF3IP1 | 0.587677 | 4.047602 | 8.87E-05 | 0.03722 |
| AC004923.1 | 1.334479 | 4.069062 | 9.02E-05 | 0.03722 |
| STARD10 | 0.73728 | 4.04237 | 9.05E-05 | 0.03722 |
| AC104809.2 | 2.117922 | 4.10527 | 9.1E-05 | 0.03722 |
| TLR2 | -0.69105 | -4.03965 | 9.15E-05 | 0.03722 |
| IL1RN | -1.39419 | -4.03757 | 9.22E-05 | 0.03722 |
| KIAA1549 | 1.458768 | 4.033994 | 9.34E-05 | 0.03722 |
| Z97634.1 | 0.690672 | 4.033826 | 9.35E-05 | 0.03722 |
| MARC2 | 0.75325 | 4.033179 | 9.37E-05 | 0.03722 |

| Table S2. E6 splicing Significantly Correlated lncRNAs.(top100) | | | | |
| --- | --- | --- | --- | --- |
| Tag | logFC | t | p-Value | adjust.p-Value |
| NR2F2-AS1 | 1.493185 | 4.741826 | 5.59E-06 | 0.02117 |
| LINC02600 | 1.864633 | 4.537691 | 1.29E-05 | 0.023273 |
| LINC01503 | -0.98219 | -4.3772 | 2.46E-05 | 0.023273 |
| CLCA4-AS1 | -1.38476 | -4.43333 | 2.3E-05 | 0.023273 |
| LINC02031 | -1.50991 | -3.98924 | 0.000125 | 0.095094 |
| AATBC | -1.10258 | -3.90175 | 0.000153 | 0.096535 |
| CPNE8-AS1 | -1.21059 | -3.79835 | 0.000226 | 0.10479 |
| LINC01679 | -1.10858 | -3.76873 | 0.000248 | 0.10479 |
| SH3PXD2A-AS1 | -1.39273 | -3.75072 | 0.000265 | 0.10479 |
| ZBED3-AS1 | 0.886763 | 3.697165 | 0.000321 | 0.10479 |
| LINC00640 | -1.48339 | -3.66075 | 0.000372 | 0.10479 |
| DIAPH2-AS1 | -1.23533 | -3.64191 | 0.000403 | 0.10479 |
| LINC00888 | 0.891726 | 3.629639 | 0.000408 | 0.10479 |
| LINC01767 | 1.018959 | 3.652255 | 0.000407 | 0.10479 |
| BMPR1B-DT | 2.313654 | 3.67729 | 0.000419 | 0.10479 |
| LINC02178 | -1.39923 | -3.6061 | 0.000458 | 0.10479 |
| MCF2L-AS1 | 1.625624 | 3.580259 | 0.000498 | 0.10479 |
| HNF1A-AS1 | 2.042818 | 3.598172 | 0.000498 | 0.10479 |
| HAS2-AS1 | -1.46998 | -3.54098 | 0.000564 | 0.109225 |
| SEPTIN4-AS1 | 0.776059 | 3.47478 | 0.000703 | 0.126842 |
| LINC01996 | 1.361005 | 3.680982 | 0.000577 | 0.109225 |
| BCAR3-AS1 | 1.370674 | 3.412845 | 0.000868 | 0.131635 |
| LINC01752 | -1.05978 | -3.40938 | 0.000873 | 0.131635 |
| BAIAP2-DT | 0.609941 | 3.397586 | 0.000904 | 0.131635 |
| MRPL23-AS1 | 1.401253 | 3.447065 | 0.0009 | 0.131635 |
| LINC01975 | 1.211982 | 3.543258 | 0.000832 | 0.131635 |
| DPYD-AS1 | -0.75767 | -3.38392 | 0.001039 | 0.140585 |
| SNAP25-AS1 | 1.125094 | 3.379953 | 0.001128 | 0.144689 |
| LINC01719 | 0.849952 | 3.30714 | 0.001223 | 0.1495 |
| LINC02595 | -0.87822 | -3.28449 | 0.001328 | 0.153415 |
| LINC00543 | 1.451843 | 3.291156 | 0.001351 | 0.153415 |
| TCEAL3-AS1 | 0.775397 | 3.427638 | 0.001146 | 0.144689 |
| FZD4-DT | 0.757489 | 3.266276 | 0.001403 | 0.153415 |
| LINC00519 | -1.39494 | -3.26569 | 0.001417 | 0.153415 |
| LINC01913 | 2.063205 | 3.293185 | 0.001553 | 0.159718 |
| LINC01778 | 1.156807 | 3.246518 | 0.001641 | 0.159718 |
| LINC02749 | 0.807607 | 3.2064 | 0.001759 | 0.159718 |
| RASSF8-AS1 | 0.75306 | 3.192687 | 0.00177 | 0.159718 |
| ARHGEF2-AS1 | 1.05308 | 3.250032 | 0.001763 | 0.159718 |
| OVOL1-AS1 | -1.04184 | -3.18349 | 0.001853 | 0.163268 |
| LINC01833 | 2.195991 | 3.181289 | 0.001942 | 0.167226 |
| SIX3-AS1 | 2.531388 | 3.324272 | 0.001699 | 0.159718 |
| LINC02560 | -1.13226 | -3.12141 | 0.002228 | 0.180106 |
| LINC01836 | 1.155216 | 3.120744 | 0.002236 | 0.180106 |
| LINC00908 | 1.109601 | 3.113858 | 0.002282 | 0.180106 |
| BCYRN1 | 0.966119 | 3.100443 | 0.002373 | 0.183216 |
| EPCAM-DT | 1.0622 | 3.092047 | 0.002476 | 0.183216 |
| LINC01091 | 1.21392 | 3.091444 | 0.002472 | 0.183216 |
| MIR4713HG | -1.00938 | -3.09057 | 0.002551 | 0.183216 |
| OBI1-AS1 | 0.644356 | 3.063205 | 0.002717 | 0.183216 |
| LINC01143 | 1.290184 | 3.06698 | 0.002808 | 0.183216 |
| P3H2-AS1 | -1.27299 | -3.05756 | 0.00283 | 0.183216 |
| LINC00520 | -1.47583 | -3.05365 | 0.002829 | 0.183216 |
| ADCY6-DT | 0.990826 | 3.0503 | 0.002787 | 0.183216 |
| LINC02677 | -1.37187 | -3.05092 | 0.002853 | 0.183216 |
| L3MBTL2-AS1 | -0.68153 | -3.04411 | 0.002832 | 0.183216 |
| LINC01341 | 0.901865 | 3.020408 | 0.003047 | 0.192449 |
| PPP1R14B-AS1 | -0.6833 | -3.00909 | 0.003151 | 0.19573 |
| MLIP-IT1 | -1.06785 | -3.0059 | 0.003273 | 0.198493 |
| DRAIC | 0.853373 | 2.996764 | 0.0033 | 0.198493 |
| LINC00482 | 1.084888 | 2.990755 | 0.003382 | 0.200216 |
| LINC02463 | 1.072941 | 3.373034 | 0.002053 | 0.172833 |
| LINC01342 | 1.079608 | 2.988169 | 0.003515 | 0.201656 |
| MNX1-AS2 | 1.157479 | 2.978008 | 0.003575 | 0.201656 |
| LINC02614 | 0.649784 | 2.97275 | 0.003522 | 0.201656 |
| SMILR | -1.05016 | -2.97641 | 0.003619 | 0.201656 |
| PCAT14 | 1.836988 | 3.011666 | 0.003694 | 0.202871 |
| KANSL1L-AS1 | 0.586415 | 2.946489 | 0.003814 | 0.206447 |
| LINC01667 | 4.240681 | 3.555102 | 0.001592 | 0.159718 |
| ALG13-AS1 | 0.715185 | 2.935603 | 0.004067 | 0.214004 |
| NR4A1AS | 1.046335 | 2.91167 | 0.004247 | 0.216363 |
| TPM1-AS | 0.853014 | 2.911315 | 0.004303 | 0.216363 |
| BCAR4 | 2.068475 | 2.945629 | 0.004387 | 0.216363 |
| TMEM72-AS1 | 0.605599 | 2.907985 | 0.004454 | 0.216363 |
| LINC02688 | 1.372245 | 2.902157 | 0.00441 | 0.216363 |
| LINC00954 | 0.855047 | 2.896139 | 0.004438 | 0.216363 |
| HOXB-AS1 | 0.695612 | 2.882892 | 0.004622 | 0.218862 |
| FOXP4-AS1 | 0.892845 | 2.867685 | 0.004847 | 0.219303 |
| CALML3-AS1 | -1.53658 | -2.86589 | 0.004862 | 0.219303 |
| TDRKH-AS1 | 0.597436 | 2.848064 | 0.005119 | 0.225545 |
| TTC39A-AS1 | 1.055927 | 2.839163 | 0.005395 | 0.234975 |
| CRPPA-AS1 | 0.655926 | 2.830495 | 0.005825 | 0.244293 |
| MIR222HG | -0.6997 | -2.81644 | 0.005618 | 0.241912 |
| LINC01474 | 0.892084 | 2.81481 | 0.006172 | 0.244293 |
| LINC02145 | 0.856404 | 2.793292 | 0.006073 | 0.244293 |
| NCMAP-DT | 1.147355 | 2.816708 | 0.006439 | 0.244678 |
| SLC44A3-AS1 | 0.67491 | 2.78789 | 0.006107 | 0.244293 |
| ARHGEF38-IT1 | 1.23022 | 2.829825 | 0.006482 | 0.244678 |
| FAM242C | 0.698925 | 2.778312 | 0.006434 | 0.244678 |
| OXCT1-AS1 | 0.612378 | 2.775392 | 0.006346 | 0.244678 |
| MIR205HG | -1.46998 | -2.76179 | 0.006587 | 0.244678 |
| EML4-AS1 | -0.73726 | -2.75019 | 0.006885 | 0.25327 |
| LRP4-AS1 | 0.599174 | 2.738861 | 0.007043 | 0.254823 |
| HOXC-AS3 | 1.130924 | 2.735719 | 0.007266 | 0.256095 |
| LINC02765 | 1.077451 | 2.71914 | 0.007515 | 0.256095 |
| LINC00327 | 0.789515 | 2.717635 | 0.007515 | 0.256095 |
| UNC5B-AS1 | -1.00426 | -2.71736 | 0.007483 | 0.256095 |
| LINC01010 | -0.90406 | -2.70868 | 0.007709 | 0.258483 |
| LINC01843 | 1.197911 | 2.703119 | 0.007918 | 0.260891 |
| IL12A-AS1 | 0.713422 | 2.695672 | 0.008086 | 0.261078 |

| Table S3. E6 splicing Significantly Correlated miRNAs. | | | |  |
| --- | --- | --- | --- | --- |
| Tag | logFC | t | *p*-Value | adjust.*p*-Value |
| hsa-miR-222-3p | -0.72978 | -4.03654 | 9.19E-05 | 0.120539 |
| hsa-miR-221-3p | -0.7176 | -3.91094 | 0.000147 | 0.120539 |
| hsa-miR-3200-3p | 1.01389 | 3.835489 | 0.000195 | 0.120539 |
| hsa-miR-6761-5p | 0.901226 | 3.670201 | 0.000392 | 0.159865 |
| hsa-miR-944 | -1.93547 | -3.61671 | 0.000432 | 0.159865 |
| hsa-miR-196a-5p | 1.184137 | 3.530566 | 0.000573 | 0.176907 |
| hsa-miR-205-3p | -0.97503 | -3.48348 | 0.00069 | 0.182569 |
| hsa-miR-3680-5p | 0.917803 | 3.649574 | 0.000883 | 0.204251 |
| hsa-miR-615-3p | 1.175801 | 3.343287 | 0.001114 | 0.229029 |
| hsa-miR-3170 | 0.719833 | 3.272498 | 0.001412 | 0.2448 |
| hsa-miR-5588-3p | 0.696195 | 3.31814 | 0.001684 | 0.2448 |
| hsa-miR-6510-3p | -1.44781 | -3.2009 | 0.001732 | 0.2448 |
| hsa-miR-375-3p | 1.63284 | 3.161524 | 0.001951 | 0.2448 |
| hsa-miR-1224-5p | 1.338657 | 3.206541 | 0.00197 | 0.2448 |
| hsa-miR-10a-3p | 0.960199 | 3.158413 | 0.001984 | 0.2448 |
| hsa-miR-1908-3p | 1.042755 | 3.235005 | 0.002788 | 0.303543 |
| hsa-miR-222-5p | -0.64292 | -3.04813 | 0.002788 | 0.303543 |
| hsa-miR-9-5p | -0.87845 | -2.92195 | 0.0041 | 0.42164 |
| hsa-miR-3171 | -3.00834 | -4.21782 | 0.004492 | 0.422985 |
| hsa-miR-194-5p | 0.982155 | 2.885733 | 0.00457 | 0.422985 |
| hsa-miR-7705 | -0.59889 | -2.79039 | 0.006152 | 0.473771 |
| hsa-miR-3651 | -0.7053 | -2.78308 | 0.006217 | 0.473771 |
| hsa-miR-194-3p | 1.05451 | 2.756282 | 0.006696 | 0.473771 |
| hsa-miR-511-5p | -0.57638 | -2.74129 | 0.006985 | 0.473771 |
| hsa-miR-6868-3p | -0.61641 | -2.76605 | 0.007448 | 0.473771 |
| hsa-miR-205-5p | -1.3504 | -2.69657 | 0.007929 | 0.473771 |
| hsa-miR-30c-2-3p | 0.732951 | 2.691795 | 0.008038 | 0.473771 |
| hsa-miR-5586-5p | -0.58086 | -2.68776 | 0.008168 | 0.473771 |
| hsa-miR-155-5p | -0.58322 | -2.686 | 0.008171 | 0.473771 |
| hsa-miR-181c-3p | 0.44571 | 2.681845 | 0.008267 | 0.473771 |
| hsa-miR-7-2-3p | 1.069403 | 2.929294 | 0.008411 | 0.473771 |
| hsa-miR-1180-3p | 0.559045 | 2.672775 | 0.008482 | 0.473771 |
| hsa-miR-196b-5p | 0.398021 | 2.664167 | 0.008691 | 0.473771 |
| hsa-miR-544b | 1.110362 | 2.963749 | 0.008868 | 0.473771 |
| hsa-miR-577 | 1.108689 | 2.658219 | 0.008958 | 0.473771 |
| hsa-miR-510-3p | -4.94154 | -4.20259 | 0.010108 | 0.519702 |
| hsa-miR-676-5p | -0.77963 | -2.67289 | 0.010538 | 0.527183 |
| hsa-miR-192-5p | 0.927109 | 2.575861 | 0.011111 | 0.541247 |
| hsa-miR-6733-3p | -0.5519 | -2.5671 | 0.011823 | 0.561139 |
| hsa-miR-29b-3p | -0.40931 | -2.5105 | 0.013277 | 0.581645 |
| hsa-miR-513c-3p | -4.54678 | -3.86686 | 0.013796 | 0.581645 |
| hsa-miR-592 | 0.714234 | 2.495992 | 0.013826 | 0.581645 |
| hsa-miR-24-2-5p | -0.39488 | -2.48421 | 0.014249 | 0.581645 |
| hsa-miR-24-1-5p | -0.39576 | -2.47613 | 0.014561 | 0.581645 |
| hsa-miR-4768-5p | 0.482574 | 2.513957 | 0.014566 | 0.581645 |
| hsa-miR-200c-5p | 0.515686 | 2.473765 | 0.014663 | 0.581645 |
| hsa-miR-1228-3p | -0.51087 | -2.46511 | 0.015154 | 0.581645 |
| hsa-miR-766-3p | 0.468011 | 2.460877 | 0.015165 | 0.581645 |
| hsa-miR-6781-3p | 0.554655 | 2.490032 | 0.015397 | 0.581645 |
| hsa-miR-5702 | 0.842776 | 2.644376 | 0.016178 | 0.598913 |
| hsa-miR-192-3p | 0.937835 | 2.425391 | 0.016725 | 0.60612 |
| hsa-miR-18b-3p | 0.640287 | 2.429501 | 0.017028 | 0.60612 |
| hsa-miR-338-5p | 0.612007 | 2.406547 | 0.017526 | 0.610224 |
| hsa-miR-6895-3p | 0.89125 | 2.554472 | 0.017856 | 0.610224 |
| hsa-miR-10a-5p | 0.65542 | 2.392307 | 0.018167 | 0.610224 |
| hsa-miR-1266-5p | 0.714642 | 2.386133 | 0.018462 | 0.610224 |
| hsa-miR-191-3p | 0.538717 | 2.371512 | 0.019273 | 0.616234 |
| hsa-miR-378d | -0.529 | -2.37329 | 0.019309 | 0.616234 |
| hsa-miR-7155-5p | -0.57567 | -2.37313 | 0.021325 | 0.669019 |
| hsa-miR-3136-5p | 0.489071 | 2.323666 | 0.02204 | 0.679946 |
| hsa-miR-1284 | 0.52397 | 2.323497 | 0.022705 | 0.688966 |
| hsa-miR-4725-3p | -0.78013 | -2.36665 | 0.023354 | 0.697242 |
| hsa-miR-29b-1-5p | -0.45368 | -2.28679 | 0.023817 | 0.697384 |
| hsa-miR-92b-3p | 0.515332 | 2.280242 | 0.024214 | 0.697384 |
| hsa-miR-146a-5p | -0.5005 | -2.27575 | 0.024489 | 0.697384 |
| hsa-miR-224-3p | -0.52404 | -2.26389 | 0.025242 | 0.707934 |
| hsa-miR-22-5p | -0.3636 | -2.24927 | 0.026169 | 0.722974 |
| hsa-miR-6503-5p | -0.56828 | -2.2469 | 0.028017 | 0.762637 |
| hsa-miR-4700-5p | 0.689129 | 2.259496 | 0.031123 | 0.815875 |
| hsa-miR-6790-3p | 2.072403 | 2.632427 | 0.031395 | 0.815875 |
| hsa-miR-6818-5p | 0.571069 | 2.262645 | 0.031431 | 0.815875 |
| hsa-miR-99b-3p | 0.451655 | 2.167873 | 0.031981 | 0.815875 |
| hsa-miR-99b-5p | 0.342172 | 2.165315 | 0.032181 | 0.815875 |
| hsa-miR-203a-3p | -0.81133 | -2.15446 | 0.033039 | 0.815875 |
| hsa-miR-744-3p | 0.438027 | 2.153213 | 0.033139 | 0.815875 |
| hsa-miR-324-3p | 0.360485 | 2.148655 | 0.033506 | 0.815875 |
| hsa-miR-4664-3p | 0.57232 | 2.147 | 0.034323 | 0.815875 |
| hsa-miR-27b-5p | -0.3488 | -2.13538 | 0.034596 | 0.815875 |
| hsa-miR-653-3p | 0.5553 | 2.16546 | 0.034821 | 0.815875 |
| hsa-miR-6821-3p | 1.046346 | 2.279915 | 0.037036 | 0.851876 |
| hsa-miR-4501 | 1.131614 | 2.196959 | 0.037278 | 0.851876 |
| hsa-miR-508-5p | -0.94697 | -2.11215 | 0.039179 | 0.884397 |
| hsa-miR-3199 | -0.36741 | -2.06563 | 0.040887 | 0.887609 |
| hsa-miR-2117 | -1.2952 | -2.40094 | 0.041023 | 0.887609 |
| hsa-miR-342-3p | -0.38605 | -2.06128 | 0.04126 | 0.887609 |
| hsa-miR-4423-5p | 0.785088 | 2.075841 | 0.042545 | 0.887609 |
| hsa-miR-28-5p | 0.222088 | 2.047141 | 0.04265 | 0.887609 |
| hsa-miR-181d-5p | 0.313897 | 2.047082 | 0.042655 | 0.887609 |
| hsa-miR-4727-5p | 0.512753 | 2.081298 | 0.043754 | 0.887609 |
| hsa-miR-584-3p | -0.63162 | -2.0521 | 0.044217 | 0.887609 |
| hsa-miR-23b-5p | -0.43163 | -2.02749 | 0.044711 | 0.887609 |
| hsa-miR-1301-3p | 0.372443 | 2.024168 | 0.044992 | 0.887609 |
| hsa-miR-147b-3p | -0.51328 | -2.01721 | 0.04595 | 0.887609 |
| hsa-miR-15b-5p | 0.314242 | 2.006241 | 0.046896 | 0.887609 |
| hsa-miR-365b-3p | -0.32539 | -2.00222 | 0.047333 | 0.887609 |
| hsa-miR-365a-3p | -0.32504 | -2.00043 | 0.047527 | 0.887609 |
| hsa-miR-301a-5p | 0.405859 | 2.000357 | 0.047568 | 0.887609 |
| hsa-miR-362-3p | -0.36019 | -1.99614 | 0.048014 | 0.887609 |
| hsa-miR-27a-3p | -0.29432 | -1.99132 | 0.048532 | 0.887609 |
| hsa-miR-5581-3p | 0.41675 | 2.003384 | 0.048627 | 0.887609 |
| hsa-miR-524-5p | 0.798918 | 2.136742 | 0.049972 | 0.887609 |

| Table S4. E6 splicing Significantly Correlated mRNAs. (top100) | | | | |
| --- | --- | --- | --- | --- |
| Tag | logFC | t | P.Value | adjust.*p*-Value |
| SYTL1 | -1.09879 | -5.58475 | 1.34E-07 | 0.002562 |
| IFI16 | -1.17295 | -5.285 | 5.23E-07 | 0.003698 |
| PRAME | 3.592019 | 5.288752 | 5.78E-07 | 0.003698 |
| TLX2 | -1.45246 | -5.07569 | 1.33E-06 | 0.005122 |
| CDON | 1.131874 | 4.817905 | 4.05E-06 | 0.009179 |
| CATSPERB | 1.245191 | 4.81468 | 4.08E-06 | 0.009179 |
| HSPB1 | -1.0659 | -4.80358 | 4.27E-06 | 0.009179 |
| ZFR2 | -2.17198 | -4.78793 | 4.63E-06 | 0.009179 |
| NBEA | 1.893135 | 4.701404 | 6.56E-06 | 0.009179 |
| ADGRD2 | -1.93058 | -4.70817 | 6.60E-06 | 0.009179 |
| SH3BP1 | -0.69449 | -4.6769 | 7.27E-06 | 0.009179 |
| RAD51AP2 | -1.7013 | -4.69841 | 7.54E-06 | 0.009179 |
| PRKAA2 | 2.041205 | 4.646675 | 8.30E-06 | 0.009179 |
| ACADL | 2.281229 | 4.644182 | 9.04E-06 | 0.009179 |
| TMPRSS11A | -3.69927 | -4.63777 | 9.08E-06 | 0.009179 |
| SLC29A4 | 1.318114 | 4.623213 | 9.08E-06 | 0.009179 |
| SIX3 | 3.04662 | 4.684888 | 9.09E-06 | 0.009179 |
| NCMAP | 2.099696 | 4.593713 | 1.03E-05 | 0.009336 |
| PLEKHA5 | 0.652799 | 4.591919 | 1.03E-05 | 0.009336 |
| C9orf152 | 2.643574 | 4.587904 | 1.07E-05 | 0.009336 |
| ASB2 | -1.32726 | -4.57148 | 1.12E-05 | 0.009378 |
| CHPT1 | 0.86728 | 4.550059 | 1.23E-05 | 0.009426 |
| LRRC6 | 1.297661 | 4.547844 | 1.24E-05 | 0.009426 |
| DUOXA1 | -1.52357 | -4.5403 | 1.28E-05 | 0.009426 |
| AGAP1 | 0.709944 | 4.524143 | 1.36E-05 | 0.009695 |
| ATP7B | 1.805513 | 4.498212 | 1.52E-05 | 0.00987 |
| KLHL21 | -0.73494 | -4.4958 | 1.53E-05 | 0.00987 |
| CHKA | 0.764118 | 4.493845 | 1.54E-05 | 0.00987 |
| KIRREL3 | 1.296173 | 4.485152 | 1.61E-05 | 0.00995 |
| EYS | 0.657658 | 4.471837 | 1.70E-05 | 0.010172 |
| CLIC6 | 2.397106 | 4.449043 | 1.85E-05 | 0.010755 |
| DUSP7 | -0.97857 | -4.41546 | 2.12E-05 | 0.011638 |
| GNA15 | -0.94992 | -4.40916 | 2.17E-05 | 0.011638 |
| ASCL5 | 1.311372 | 4.406163 | 2.30E-05 | 0.011638 |
| DRD2 | 2.120823 | 4.409887 | 2.30E-05 | 0.011638 |
| CDH7 | 2.549672 | 4.54959 | 2.44E-05 | 0.011638 |
| ANXA8L1 | -2.21933 | -4.3793 | 2.45E-05 | 0.011638 |
| AIM2 | -2.14561 | -4.36327 | 2.61E-05 | 0.011638 |
| KCNQ3 | 1.374156 | 4.341764 | 2.84E-05 | 0.011916 |
| OSBPL10 | 0.735083 | 4.341508 | 2.84E-05 | 0.011916 |
| INKA1 | -1.39842 | -4.34036 | 2.86E-05 | 0.011916 |
| DENND5B | 1.004126 | 4.319366 | 3.10E-05 | 0.012673 |
| STXBP6 | 2.170936 | 4.309862 | 3.25E-05 | 0.012808 |
| CLDN9 | 1.958986 | 4.304968 | 3.30E-05 | 0.012808 |
| KRT14 | -3.84534 | -4.30101 | 3.34E-05 | 0.012808 |
| CNKSR2 | 1.639768 | 4.289833 | 3.52E-05 | 0.01325 |
| PKDCC | 1.827976 | 4.26975 | 3.77E-05 | 0.013927 |
| VSNL1 | -2.36865 | -4.25581 | 3.99E-05 | 0.014111 |
| TMPRSS11D | -3.41548 | -4.25484 | 4.02E-05 | 0.014111 |
| SOBP | 1.282356 | 4.249683 | 4.08E-05 | 0.014111 |
| CENPV | 1.24584 | 4.247468 | 4.12E-05 | 0.014111 |
| S100A2 | -1.71423 | -4.23454 | 4.33E-05 | 0.014203 |
| ANXA8 | -2.16254 | -4.23404 | 4.34E-05 | 0.014203 |
| SGSM1 | 1.665177 | 4.233632 | 4.37E-05 | 0.014203 |
| PNMA2 | 1.824462 | 4.230053 | 4.47E-05 | 0.014295 |
| TMEM150C | 1.163525 | 4.214111 | 4.69E-05 | 0.014755 |
| KRT5 | -2.72564 | -4.19826 | 4.99E-05 | 0.015194 |
| NRIP3 | -0.95728 | -4.1754 | 5.45E-05 | 0.015609 |
| TPO | 1.788249 | 4.192131 | 5.47E-05 | 0.015609 |
| BTBD3 | 0.801286 | 4.171565 | 5.53E-05 | 0.015609 |
| CDC42EP1 | -0.69678 | -4.16069 | 5.77E-05 | 0.015682 |
| SAMD9 | -1.13815 | -4.16012 | 5.78E-05 | 0.015682 |
| ARHGEF38 | 1.758881 | 4.159164 | 5.80E-05 | 0.015682 |
| ERVMER34-1 | -1.26954 | -4.15268 | 5.95E-05 | 0.015856 |
| LRP5 | 0.615165 | 4.132032 | 6.44E-05 | 0.016711 |
| SEMA4G | 1.215431 | 4.13189 | 6.44E-05 | 0.016711 |
| SPRR1A | -2.94944 | -4.12748 | 6.67E-05 | 0.016795 |
| CLEC2B | -1.40167 | -4.12196 | 6.69E-05 | 0.016795 |
| SCUBE3 | 1.481643 | 4.120213 | 6.74E-05 | 0.016795 |
| FIGNL2 | 0.97543 | 4.121941 | 6.97E-05 | 0.017142 |
| GBP6 | -2.86196 | -4.09373 | 7.49E-05 | 0.017838 |
| COL25A1 | 1.605696 | 4.109458 | 7.52E-05 | 0.017838 |
| LRP6 | 0.603607 | 4.09027 | 7.56E-05 | 0.017838 |
| CAPNS2 | -2.559 | -4.09124 | 7.62E-05 | 0.017838 |
| SYNGR3 | -1.27693 | -4.07799 | 7.92E-05 | 0.018129 |
| PPM1H | 1.513128 | 4.074802 | 8.01E-05 | 0.018129 |
| IL20RB | -2.03011 | -4.07429 | 8.03E-05 | 0.018129 |
| RIN1 | -0.71969 | -4.06821 | 8.22E-05 | 0.018254 |
| GALNTL6 | 1.657394 | 4.069403 | 8.35E-05 | 0.018254 |
| TRAF3IP1 | 0.588775 | 4.063361 | 8.37E-05 | 0.018254 |
| DDX60 | -0.8448 | -4.05666 | 8.59E-05 | 0.018513 |
| SLC10A6 | -2.00645 | -4.05283 | 8.75E-05 | 0.018648 |
| SPDEF | 2.491246 | 4.043131 | 9.04E-05 | 0.01886 |
| STARD10 | 0.738378 | 4.042999 | 9.04E-05 | 0.01886 |
| KIAA1549 | 1.459866 | 4.033774 | 9.36E-05 | 0.019039 |
| IL1RN | -1.3931 | -4.03201 | 9.43E-05 | 0.019039 |
| TLR2 | -0.68996 | -4.02708 | 9.60E-05 | 0.019194 |
| PGM2L1 | 0.930878 | 4.005922 | 0.000104 | 0.020572 |
| SFN | -1.21149 | -3.99606 | 0.000108 | 0.020933 |
| BNC1 | -2.84708 | -3.99585 | 0.000108 | 0.020933 |
| FARP1 | 0.949148 | 3.993176 | 0.000109 | 0.020933 |
| SPRR3 | -3.07448 | -3.98202 | 0.000114 | 0.021488 |
| MICALL1 | -0.74753 | -3.9809 | 0.000114 | 0.021488 |
| LRRC75A | 0.874635 | 3.973287 | 0.000118 | 0.021599 |
| METTL7B | 1.696876 | 3.971778 | 0.000118 | 0.021599 |
| TMEM263 | 0.624963 | 3.958731 | 0.000124 | 0.02216 |
| C2orf72 | 2.100553 | 3.959501 | 0.000125 | 0.02216 |
| GPR150 | -0.97608 | -3.95039 | 0.000128 | 0.02216 |
| ADCY2 | 1.783379 | 3.95196 | 0.000128 | 0.02216 |
| NOL4L | 0.921005 | 3.944046 | 0.000131 | 0.022288 |

Table S5. Gene sequences and PCR primers.

**Gene sequences**

| E6 |
| --- |
| >NC_001526.4:7125-7601 Human papillomavirus type 16, complete genome  GGATCCGCCACCATGCACCAAAAGAGAACTGCAATGTTTCAGGACCCACAGGAGCGACCCAGAAAGTTACCACAGTTATGCACAGAGCTGCAAACAACTATACATGATATAATATTAGAATGTGTGTACTGCAAGCAACAGTTACTGCGACGTGAGGTATATGACTTTGCTTTTCGGGATTTATGCATAGTATATAGAGATGGGAATCCATATGCTGTATGTGATAAATGTTTAAAGTTTTATTCTAAAATTAGTGAGTATAGACATTATTGTTATAGTTTGTATGGAACAACATTAGAACAGCAATACAACAAACCGTTGTGTGATTTGTTAATTAGGTGTATTAACTGTCAAAAGCCACTGTGTCCTGAAGAAAAGCAAAGACATCTGGACAAAAAGCAAAGATTCCATAATATAAGGGGTCGGTGGACCGGTCGATGTATGTCTTGTTGCAGATCATCAAGAACACGTAGAGAAACCCAGCTGTAAGAATTC |

E6*

| >NC_001526.4:7125-7459 Human papillomavirus type 16, complete genome ATGCACCAAAAGAGAACTGCAATGTTTCAGGACCCACAGGAGCGACCCAGAAAGTTACCACAGTTATGCACAGAGCTGCAAACAACTATACATGATATAATATTAGAATGTGTGTACTGCAAGCAACAGTTACTGCGACGTGAGGTATATGACTTTGCTTTTCGGGATTTATGCATAGTATATAGAGATGGGAATCCATATGCTGTATGTGATAAATGTTTAAAGTTTTATTCTAAAATTAGTGAGTATAGACATTATTGTTATAGTTTGTATGGAACAACATTAGAACAGCAATACAACAAACCGTTGTGTGATTTGTTAATTAGGTGTATTAA |
| --- |

| **PCR-Primers** | |  |
| --- | --- | --- |
|  | sense（5'-3'） | antisense（5'-3'） |
| E6 | GAACAGCAATACAACAAACCGT | TCAGGACACAGTGGCTTTTGA |
| E6* | CAGGAGCGACCCAGAAAGTT | GCAGTAACTGTTGCTTGCAGT |
| CXCL2 | CTGCGCCCAAACCGAAGTCATA | TTCAGGAACAGCCACCAATAAGC |
| SDC1 | CTGCCGCAAATTGTGGCTAC | TGAGCCGGAGAAGTTGTCAGA |
| PRLR | TCTCCACCTACCCTGATTGAC | CGAACCTGGACAAGGTATTTCTG |
| GAPDH | CTCCTCCTGTTCGACAGTCAGC | CCCAATACGACCAAATCCGTT |
